# DIAPH1-Deficiency is Associated with Major T, NK and ILC Defects in Humans

**DOI:** 10.1007/s10875-024-01777-8

**Published:** 2024-08-09

**Authors:** Zehra Busra Azizoglu, Royala Babayeva, Zehra Sule Haskologlu, Mustafa Burak Acar, Serife Ayaz-Guner, Fatma Zehra Okus, Mohammad Bilal Alsavaf, Salim Can, Kemal Erdem Basaran, Mehmed Fatih Canatan, Alper Ozcan, Hasret Erkmen, Can Berk Leblebici, Ebru Yilmaz, Musa Karakukcu, Mehmet Kose, Ozlem Canoz, Ahmet Özen, Elif Karakoc-Aydiner, Serdar Ceylaner, Gülsüm Gümüş, Huseyin Per, Hakan Gumus, Halit Canatan, Servet Ozcan, Figen Dogu, Aydan Ikinciogullari, Ekrem Unal, Safa Baris, Ahmet Eken

**Affiliations:** 1 Department of Medical Biology, Faculty of Medicine, Erciyes University, Kayseri, 38039 Türkiye; 2 Genome and Stem Cell Center, Kayseri, 38039 Türkiye; 3 The Istanbul Jeffrey Modell Diagnostic Center for Primary Immunodeficiency Diseases, The Isil Berat Barlan Center for Translational Medicine, Division of Pediatric Allergy and Immunology, Department of Pediatrics, Faculty of Medicine, Marmara University, Istanbul, Türkiye; 4 Division of Pediatric Allergy and Immunology, Department of Pediatrics, Faculty of Medicine, Ankara University, Ankara, Türkiye; 5 Department of Molecular Biology and Genetics, Izmir Institute of Technology, Izmir, Türkiye; 6 Erciyes University Medical School, Kayseri, Türkiye; 7 Department of Physiology, Faculty of Medicine, Erciyes University, Kayseri, 38039 Turkey; 8 Division of Pediatric Hematology and Oncology, Department of Pediatrics, Faculty of Medicine, Erciyes University, Kayseri, 38039 Turkey; 9 Department of Medical Genetics, Ankara University Faculty of Medicine, Ankara, Türkiye; 10 Division of Pediatric Pulmonology, Department of Pediatrics, Faculty of Medicine, Erciyes University, Kayseri, 38039 Türkiye; 11 Department of Pathology, Faculty of Medicine, Erciyes University, 38039 Kayseri, Türkiye; 12 Intergen, Genetic, Rare and Undiagnosed Diseases, Diagnosis and Research Center, Ankara, Türkiye; 13 Division of Pediatric Radiology, Department of Radiology, Erciyes University Faculty of Medicine, Kayseri, Türkiye; 14 Division of Pediatric Neurology, Department of Pediatrics, Faculty of Medicine, Erciyes University, Kayseri, 38039 Türkiye; 15 Department of Biology, Faculty of Science, Erciyes University, Kayseri, 38039 Türkiye; 16 School of Health Sciences, Hasan Kalyoncu University, Gaziantep, Türkiye; 17 Medical Point Hospital, Pediatric Hematology Oncology and BMT Unit, Gaziantep, Türkiye

**Keywords:** DIAPH1, Macrothrombocytopenia, Immunodeficiency, Cytoskeletal defects

## Abstract

Loss of function mutations in Diaphanous related formin 1 (*DIAPH1)* are associated with seizures, cortical blindness, and microcephaly syndrome (SCBMS) and are recently linked to combined immunodeficiency. However, the extent of defects in T and innate lymphoid cells (ILCs) remain unexplored. Herein, we characterized the primary T, natural killer (NK) and helper ILCs of six patients carrying two novel loss of function mutation in *DIAPH1* and Jurkat cells after *DIAPH1* knockdown. Mutations were identified by whole exome sequencing. T-cell immunophenotyping, proliferation, migration, cytokine signaling, survival, and NK cell cytotoxicity were studied via flow cytometry-based assays, confocal microscopy, and real-time qPCR. CD4^+^ T cell proteome was analyzed by mass spectrometry. p.R351* and p.R322*variants led to a significant reduction in the DIAPH1 mRNA and protein levels. DIAPH1-deficient T cells showed proliferation, activation, as well as TCR-mediated signaling defects. DIAPH1-deficient PBMCs also displayed impaired transwell migration, defective STAT5 phosphorylation in response to IL-2, IL-7 and IL-15. In vitro generation/expansion of Treg cells from naïve T cells was significantly reduced. shRNA-mediated silencing of *DIAPH1* in Jurkat cells reduced DIAPH1 protein level and inhibited T cell proliferation and IL-2/STAT5 axis. Additionally, NK cells from patients had diminished cytotoxic activity, function and IL-2/STAT5 axis. Lastly, DIAPH1-deficient patients’ peripheral blood contained dramatically reduced numbers of all helper ILC subsets. DIAPH1 deficiency results in major functional defects in T, NK cells and helper ILCs underlining the critical role of formin DIAPH1 in the biology of those cell subsets.

**Graphical Abstract:**

**Figure:**
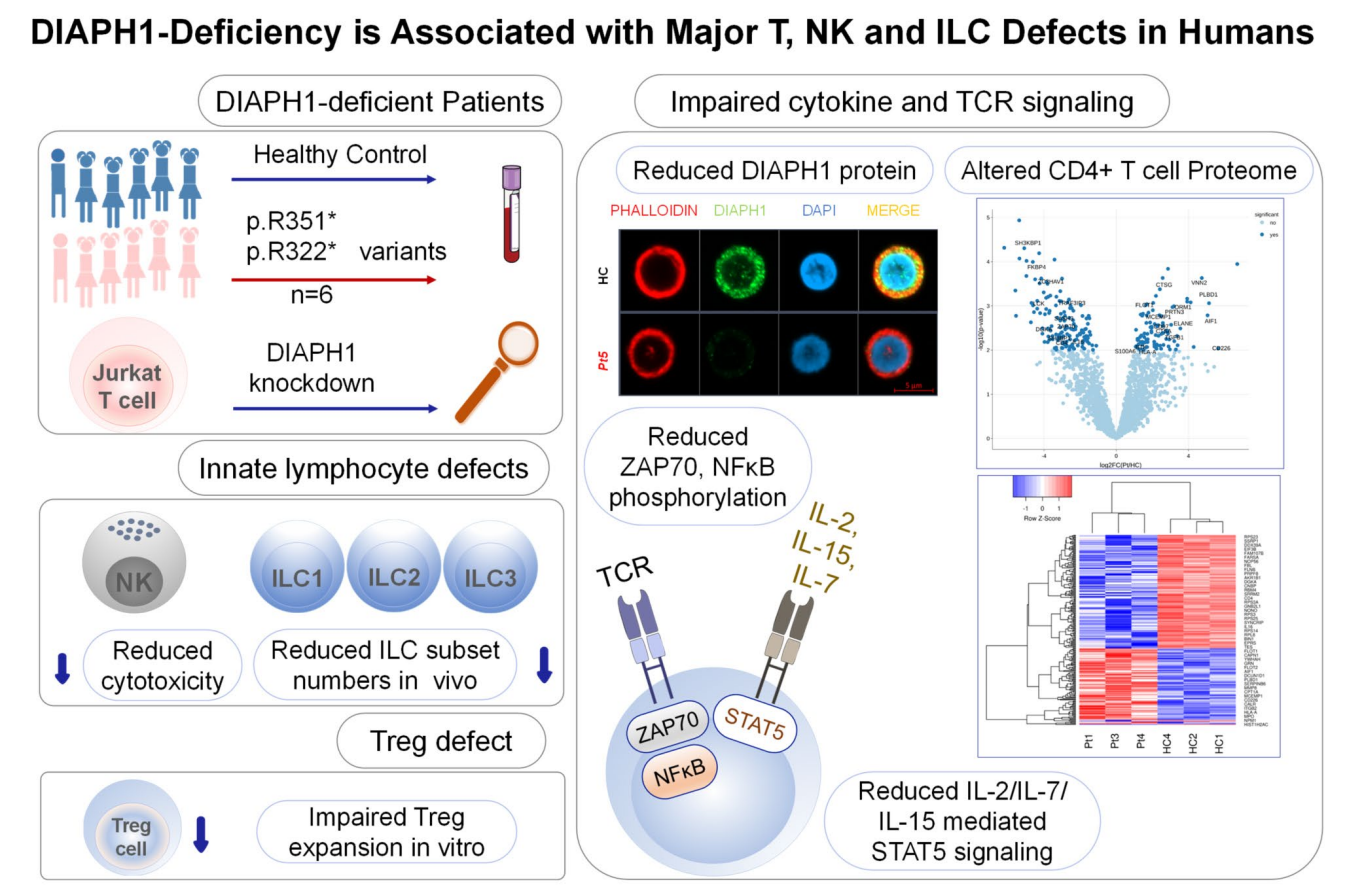
The summary of findings are presented as a graphical abstract. DIAPH1 deficiency results in multiple defects in CD4^+^ T, Treg, NK cells and ILCs.

**Supplementary Information:**

The online version contains supplementary material available at 10.1007/s10875-024-01777-8.

## Introduction

Diaphanous related formin 1 (DIAPH1) is a 140 kD ubiquitously expressed cytoskeletal protein (encoded by its gene on chromosome 5q31, which consists of 28 exons) and belongs to formin family [1–3]. In the vertabrates, including humans, 3 homologs of DIAPH proteins (Dia1-3) have been identified, which are believed to form through gene duplications [1, 2]. T cells also express the DIAPH paralogs (DIAPH1, 2 and 3), DIAPH1 being the predominantly expressed isoform of Diaphanous-related formins (DRFs) [4].

Structurally, DIAPH1 has an N-terminal GTPase binding domain (GBD), an adjacent Diaphanous inhibitory domain (DID) followed by a dimerization domain (DD), two highly conserved formin homology (FH) 1 and FH2 domains regulating actin polymerization, and a C-terminal DAD (Diaphanous autoregulatory area) domain [5]. FH1 and FH2 initiate actin assembly [6]. The FH1 domain affects the function of the actin-binding FH2 domain by binding to profilin, which is necessary for the elongation of the actin chain by the diaphanous (DIAPH1) [7–9]. The DID domain of DIAPH1, also known as FH3, recognizes the DAD at C terminus [10]. The interaction between DID and DAD domains is required for autoregulation of microtubule networks and F-actin bundles. Additional dimerization elements called DD and CC (coiled coil) contribute to the overall structural assembly of formins [10–13]. DIAPH proteins are kept inactive in the absence of extracellular stimuli [14]. In the resting state, intramolecular interaction between the C-terminal DAD and the FH3 N-terminal recognition domain blocks FH1 and FH2 domains, and DIAPH1 is auto-inhibited [10]. All formins must be activated by interacting with Rho GTPases. mDia-1 is activated by Rho (RhoA-C), while mDia-2 and mDia-3 are activated by Rho (RhoA-C) as well as by Rac and Cdc42 [7, 15, 16].

Nonredundant roles of mammalian Dia family members have recently been described in ciliogenesis and cilia maintenance. Similarly, nonredundant roles of the DIAPH1-3 proteins were described in cell motility and the capture of cortical microtubules [1, 2, 4]. In addition, formins play a role in differentiation, migration, proliferation, regulation of cell morphology and cytoskeletal organization [17–19]. DIAPH1 is critical in linear actin nucleation/polymerization and microtubule stability. It regulates the spindle formation and cell division during mitotic cell division in human neuronal precursor cells, thus has critical roles in neurodevelopmental processes [17].

Autosomal dominant mutations of DIAPH1, (possibly resulting in gain of function), were associated with deafness with or without thrombocytopenia. Loss of function mutations (LoF) in DIAPH1 are associated with seizures, cortical blindness, and microcephaly syndrome (SCBMS) [1, 17]. More recently, a study by Kaustio et al. revealed that LoF mutations in DIAPH1 resulted in combined immunodeficiency affecting both T and B cells [4]. In that study, defective-T cell activation and proliferation, mitochondrial dysfunction, and reduced B cell function and number have been described, marking mutations in DIAPH1 as causative for a novel form of combined immunodeficiency. However, whether T cell proximal signaling was affected, and how and if DIAPH1 deficiency impacted CD4^+^ T helper subsets (Th17, Treg), natural killer (NK) and innate lymphoid cell (ILC) functions and biology have not been explored in human cells thus far [4]. Although murine studies with mouse ortholog mDia1 also revealed lymphopenia, T cell activation and T cell receptor (TCR) signaling defects in two separate lines of work [20, 21], similarly in mice the potential role of DIAPH1 in CD4^+^ T helper subset generation, maintenance and function, NK cell and helper ILC biology have not been assessed to this day.

In the current study, we aimed to characterize T cells, NK cells and helper ILCs in 6 unrelated patients with two LoF mutation in DIAPH1, and the consequences of shRNA-mediated silencing of DIAPH1 in Jurkat T cells.

## Materials and Methods

### Human Sample

Peripheral blood samples were collected from the patients, relatives and healthy volunteers. All the experiments of this study were performed according to the relevant guidelines and regulations. See ***Supplementary Methods*** for detailed method description.

### Statistical Analysis

The normality/lognormality of the data was analyzed prior to tests via Shapiro Wilk test. For multiple comparisons of normally distributed data One-way ANOVA with Dunnett’s post-test analysis was used for significance analyses. Kruskal Walis with Tukey’s Post hoc test was used for multiple comparison analyses of non-normally distributed data. Pairwise comparisons were performed either with the student’s t-test or Mann-Whitney U test, depending on the normality of the data. A p-value < 0.05 is accepted as significant. Graph Pad Prism 6 software was used to analyze the data.

## Results

### Clinical and Laboratory Findings of DIAPH1-Deficient Patients

Six unrelated patients were enrolled from different clinics across Turkiye presented with symptoms of SCBMS. A summary of the demographic and clinical characteristics of the patients is given in (Table S1). Five of the patients were females, and one was male, the minimum-maximum age of the patients was (2–16 years). All patients were born to unrelated families with consanguineous marriages. The fact that the families were from distant regions of Turkiye suggested a founder effect. All of the patients had microcephaly, mental retardation, and epilepsy, thus exhibited the hallmarks of SCBMS disease [17]. All patients except Pt3 and Pt4 were blind. All patients failed to thrive, and had recurrent infections, except for P5, all had severe infections, showing symptoms of immunodeficiency. The disease symptoms started in the first year of life for all patients. Detailed immunological workups of patients are presented in Table S2. Pt1, 2, 3, and 4 had lymphopenia. All the patients had reduced CD4^+^ T cell absolute counts. Additionally, Pt3 and 4 also had lower absolute numbers of CD8^+^ T cells. Pt1 and 4 had reduced B cell number. Pt1, 3, and 4 had reduced absolute counts of NK cell. The antibodies against mumps and measles were absent in Pts 1, 2, 3, 4 and 5.

**Fig. 1:**
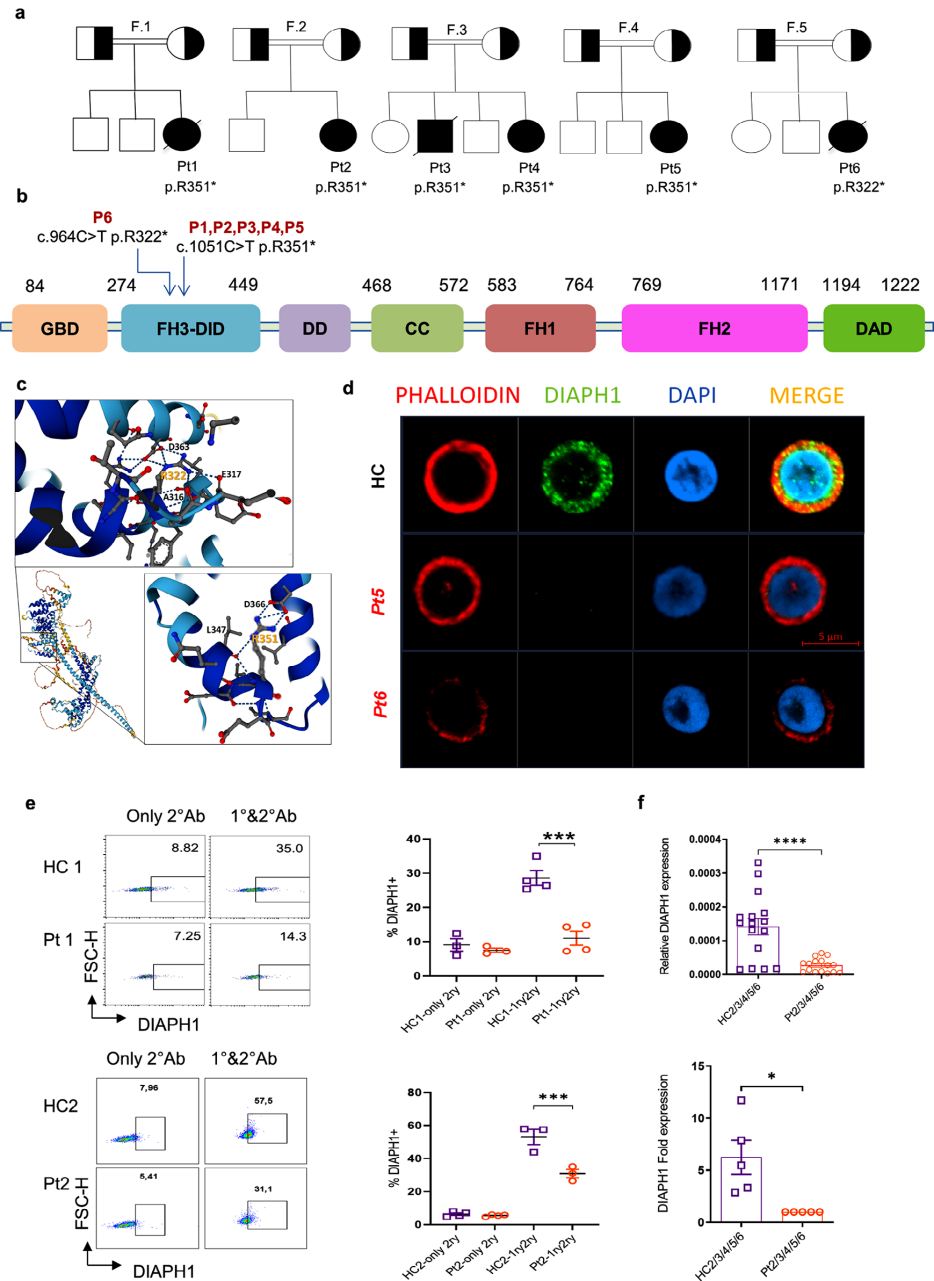
p.R351* and p.R322* mutations reduce DIAPH1 protein and mRNA expression. **(a)** Pedigree of the patients. **(b)** Cartoon of DIAPH1 domains and the mutations identified in 6 patients (Pt) enrolled in this study. **(c)** The structure of DIAPH1, position of p.R351* and p.R322* mutations. **(d)** anti-CD3/28 activated PBMCs of healthy controls and Pt 1–6 were stained with anti-DIAPH1 and DAPI. The slides were examined by confocal microscopy at 63x magnification, and two representative pictures (Pt5 and Pt6) are shown. **(e)** Flow cytometric quantification of DIAPH1 protein staining in PBMC samples from Pt1and Pt2 showed significant reduction in the protein levels. Representative flow plots and quantified bar graphs are shown. Only 2°Ab indicates no primary antibody addition and is a negative control. **(f)** The gene expression of *DIAPH1* in PBMCs was quantified by real-time qPCR, 3 technical replicates/patients (upper) and averaged graphs (bottom) were shown separately. Patient: Pt. Healthy control: HC. For *p*-values *: <0.05, ***<0.001, **** < 0.0001

Whole exome sequencing of six patients revealed a homozygous variant in the *DIAPH1* gene c.1051 C > T; p.R351* (NM_005219.4, isoform 1) of Pts1,2,3,4,5, and the c.964 C > T, p.R322* (NM_001079812.3, isoform 2) variant in Pt6. The pedigrees of the patients are illustrated in (Fig. 1a). These nonsense mutations fall in the FH3-DID and are expected to result in early termination in translation, and potential truncate proteins devoid of critical FH1 and FH2 domains (Fig. 1b, c). The mutations were confirmed by the Sanger sequencing or next-generation sequencing. The variant was identified initially using isoform 1 for Pt1,2,3,5 (NM_005219.4) and isoform 2 for Pt6 (NM_001079812.3), which are 20 aa apart.

The p.R351* and p.R322* variants have recently been listed in gnomAD (their minor allele frequencies 2.05e-6 and 3.1e-6, respectively). However, no homozygotes have been reported for these variants, and no detailed investigations or functional studies have been previously performed.

### p.R351* and p.R322* Variants Resulted in Reduced DIAPH1 Protein and mRNA Message in PBMCs

We employed confocal microscopy and flow cytometry to better understand the consequences of p.R351* and p.R322* mutations for the DIAPH1 protein expression. Confocal microscopy revealed no DIAPH1 signal in 6 of the patients’ PBMCs compared to healthy controls (Fig. 1d). Similarly, flow cytometric examination of Pt1 and 2 at different times, as well as those of Pt3 and 6 simultaneously (Fig S1a), ) revealed a significant reduction in the protein levels (Fig. 1e). We conducted RT-qPCR to compare the mRNA levels of all patients, which revealed significantly lower levels of mRNA message in the patient cells relative to the control (Figs. 1f and S1b), suggesting the involvement of nonsense-mediated mRNA decay (NMD). Collectively, these data support that both DIAPH1 variants result in protein loss and reduced mRNA levels in gene expression, arguing for their pathogenic nature.

### DIAPH1 Deficiency Results in Impaired T Cell Activation, Proliferation, and T Cell Receptor Signaling

**Fig. 2:**
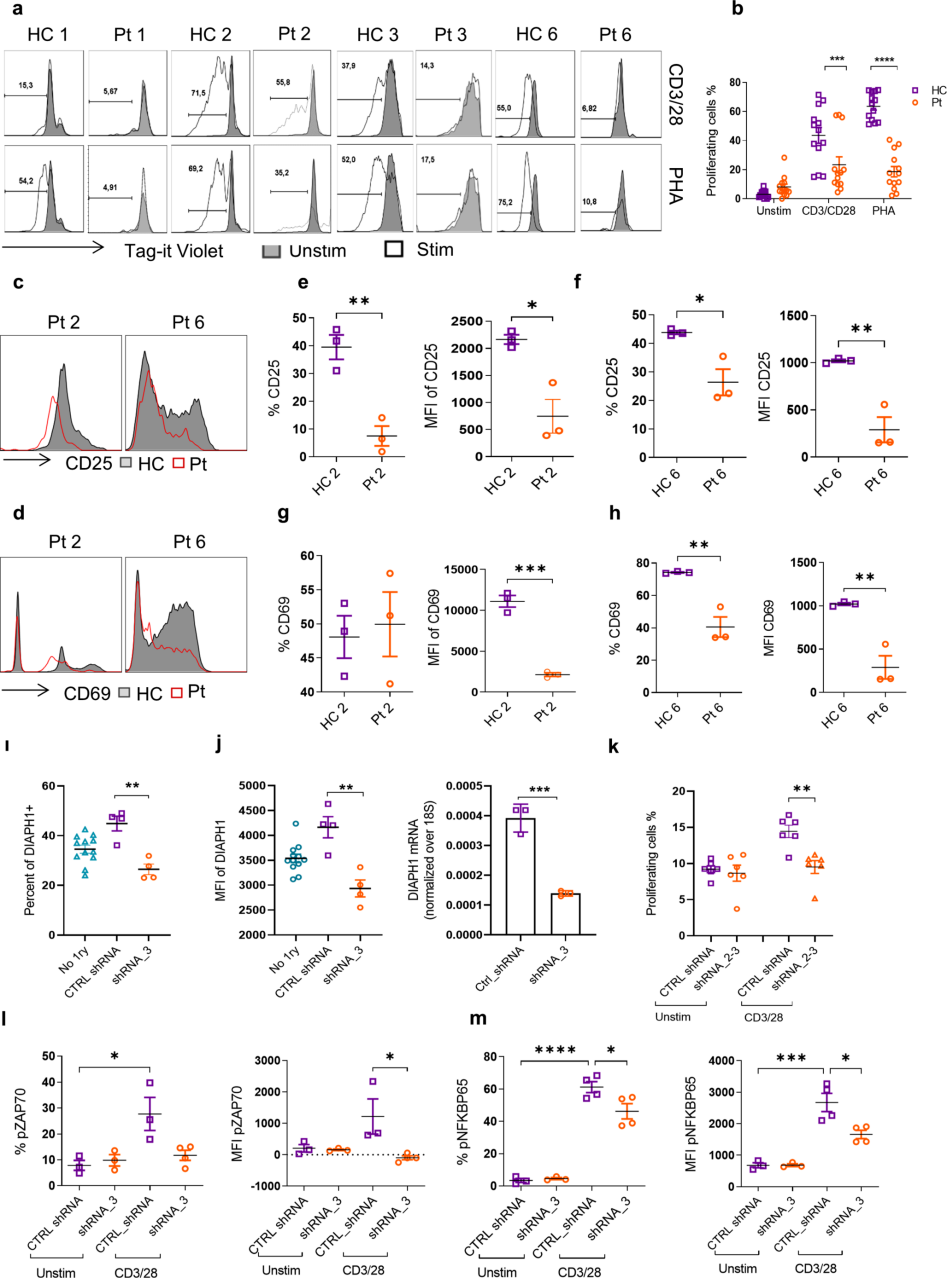
p.R351* and p.R322* variants of DIAPH1 or DIAPH1 knockdown impairs T cell activation, proliferation and T cell receptor signaling. **(a)** PBMCs from Pt1, 2, 3 and 6 were labeled with Tag-it-violet and activated with anti-CD3/CD28 or PHA for 4 days in complete medium, cell proliferation was measured by flow cytometry. Percentages of proliferating cells were shown as histogram plots, **(b)** and quantified bar graphs, three technical replicates/patient. **(c-d).** PBMCs from the Pt2, Pt6 and controls were stimulated in triplicate overnight with or without anti-CD3/CD28 and cells were stained with early activation markers CD25 and CD69. **(e-h).** Quantified graphs of CD25 and CD69 surface expression in the peripheral blood of Pt2, Pt6 and healthy controls. **(e-f)** Percentages and Mean Fluorescent intensity (MFI) of CD25 surface expression. **(g-h)** Percentages and MFI of CD69 surface expression. **(i-j)** Percentages and MFI of DIAPH1 post-silencing in Jurkat cell line. Jurkat cells infected by shRNA#3 or control shRNA were collected and DIAPH1 expression was analyzed by RT-PCR. **(k)** After DIAPH1 silencing Jurkat cells were labeled with CFSE and activated with CD3/CD28 for 3 days in complete medium. Then, the percentage of cell proliferation was quantified. **(l-m)**. After DIAPH1 silencing, Jurkat cells were activated with anti-CD3/CD28 for 30 min in serum free medium. Then, 20 µg of secondary antibody (APC anti-mouse IgG) was added and incubated for 20 min at 37ºC and finally fixed, permeabilized and stained for pZAP70, p-NF-κBp65, and p-ERK. For *p*-values *<0.05, **<0.01,***<0.001, ****< 0.0001

DIAPH1 has been previously shown to provide structural support and regulate T cell signaling pathways [4]. We investigated whether the p.R351* and p.R322* variants would cause similar defects in T cell activation, proliferation, and signaling pathways. Upon activation of T cells with various mitogens such as CD3/28 and PHA for 4 days, the proliferation of T cells was severely impaired in DIAPH1-deficient T cells (Fig. 2a, b). Additionally, the early activation markers CD25 and CD69 surface expressions were significantly reduced by DIAPH1-deficient T cells (Fig. 2c-h). The same impairments in proliferation and activation were observed when sorted CD4^+^ T cells were used (Fig. S1b**)**, suggesting a T-cell intrinsic defect.

To investigate potential issues in the TCR signaling pathway, we conducted *DIAPH1* knockdown experiments in the Jurkat T cell line using three different commercial shRNA constructs with a GFP reporter (Fig. S2a**)**. The experiments showed that shRNA#3 was particularly effective in downregulating DIAPH1 protein and mRNA (Figs. S2b-c and Fig. 2i-j) and was therefore used in the subsequent experiments. We observed a decrease in the proliferation of Jurkat cells after DIAPH1 knockdown **(**Fig. 2k). Our examination of the TCR signaling pathway revealed impaired TCR signaling components including ZAP70 and NF-κB, after the knockdown of DIAPH1 in Jurkat cells upon stimulation with anti-CD3/28 **(**Fig. 2l-m and S1d, e). ERK phosphorylation in DIAPH1-deficient Jurkat T cells was normal (Fig. S2f-g).

**Fig. 3:**
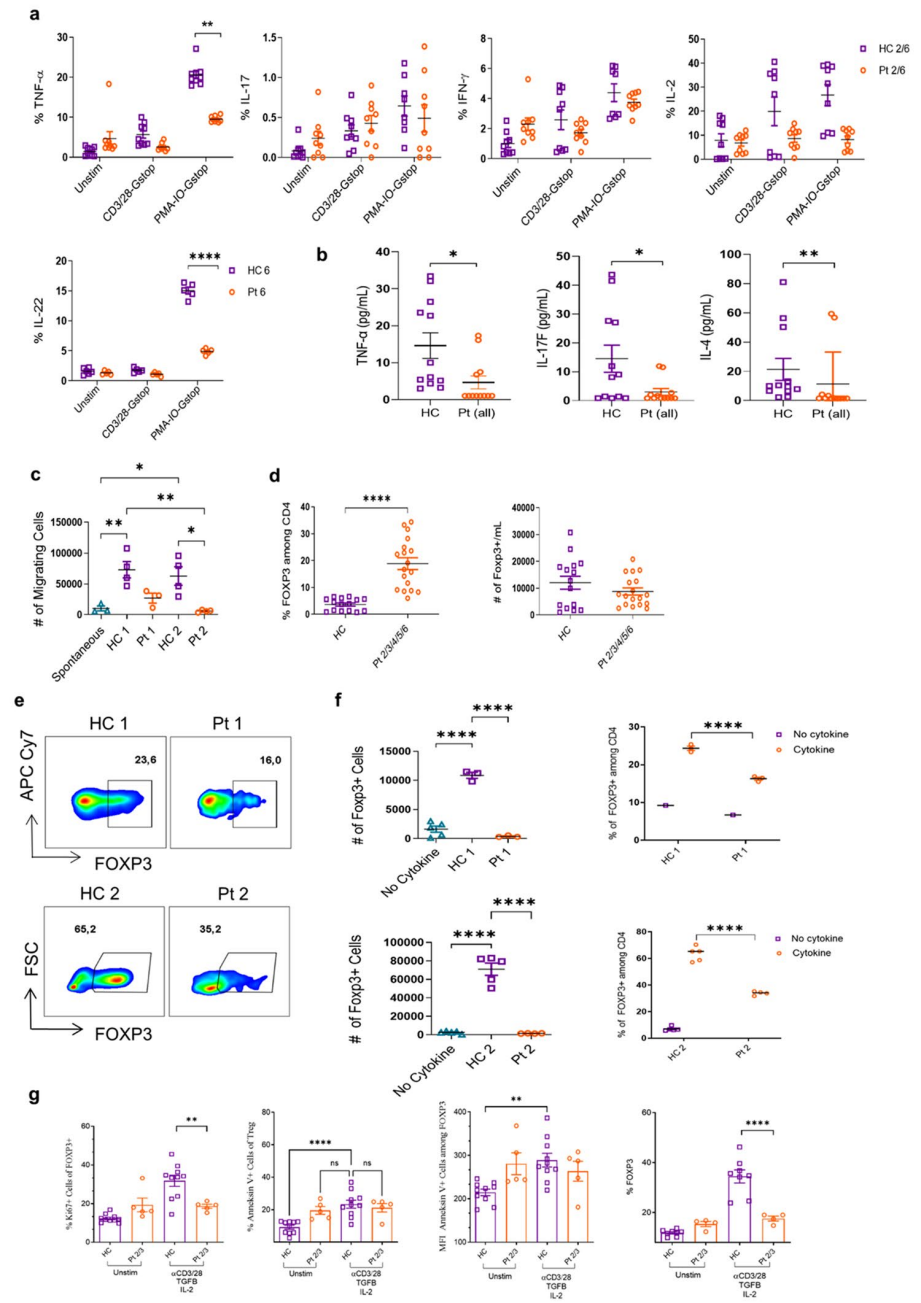
Impaired cytokine production by DIAPH1-deficient T cells, and impaired Treg cell expansion **(a-c)**. DIAPH1-deficieny does not affect the differentiation of naïve CD4^+^ T cells into Treg cells, but negatively impacts expansion of Treg cells **(d-f)**. **(a)** PBMCs from Pt2, Pt6 and healthy controls were stimulated with anti-CD3/28 or PMA/ionomycin and Golgi Stop for 4 h and percentage of IL-17, IL-22, IL-2, TNFα and IFN-γ cytokines production were quantified. **(b)** The levels of IL-17, TNFα and IL-4 in the serum of healthy controls and DIAPH1 patients were measured by ELISA. **(c)** Transwell migration of equal numbers of healthy control and Pt1/Pt2 lymphocytes into the medium with FBS was tested after 4–5 h. and quantified absolute number **(d)** The frequency of FOXP3^+^ cells among CD4+, or their absolute numbers in PMBCs of DIAPH1 patients and healthy controls was shown (5 patients, with 2–3 technical replicates). **(e-f)** Naive CD4^+^ T cells from Pt1/Pt2 and healthy controls were selected magnetically, polarized into Treg cells ex vivo and FOXP3 staining was performed. **(e)** representative flow plot. **(f)** Absolute number and the percentages of FOXP3^+^ cells in cultures. **(g)** Total CD4^+^ T cells from Pt2/Pt3 and healthy controls were selected magnetically, polarized into Treg cells ex vivo and Ki67, ANNEXINV, and FOXP3 staining was performed on day 5. For *p*-values *<0.05, **<0.01, ****< 0.0001

To better probe the functional consequences of DIAPH1 deficiency in T cells, cytokine production of T cells upon anti-CD3/CD28 or PMA/Ionomycin activation was examined. DIAPH1-deficient T cells produced significantly lower levels of TNFα and IL-22 (but not IL-17 A, IL-2 and IFN-γ, although a trend was observed) **(**Fig. 3a). These findings are consistent with a defect in the TCR signaling pathway. In line with these findings, serum levels of TNF-α, IL-17 A, and IL-4 were significantly reduced in the patients compared with those of healthy age and sex-matched controls **(**Fig. 3b**)**.

Since DIAPH1 is associated with cytoskeleton regulation, we assessed the ability of peripheral blood mononuclear cells to migrate through a trans-well chamber. Equal numbers of healthy control and patient cells were added to the FBS-free medium. The cells were then placed in 24-well plates with complete medium and FBS and cultured for 4 h to migrate to the lower wells. Our findings indicate that individuals with DIAPH1 defects exhibit lower T cell migration across the transwell **(**Fig. 3c**)**.

We also noticed that PBMCs from DIAPH1-deficient patients died more frequently in the culture medium. After stimulating the cells with anti-CD3/CD28 overnight, we confirmed that DIAPH1-deficient cells died more frequently and stained more with Annexin V and 7-AAD **(**Fig. S3a).

All these data collectively suggest that T cells with the homozygous p.R351* and p.R322* DIAPH1 variants have multiple defects in T cell proliferation, activation, survival, and migration as well as partial defects in the TCR signaling pathway. Additionally, T cells have limited cytokine production capabilities.

### DIAPH1 Deficiency Results in Impaired Treg Cell Expansion, and Impaired STAT5 Phosphorylation

As previously mentioned, cytoskeletonopathies can result in defects in CD4^+^ T cell subsets. as shown by Treg [22–30] and Th17 [31, 32] cell defects in DOCK8, CDC42 and WASP deficiencies Thus, we investigated whether DIAPH1 deficiency had an impact on regulatory T cells. Our findings revealed a significantly elevated percentage of FOXP3^+^ Treg cells in the peripheral blood of DIAPH1-deficient patients compared with healthy controls (Fig. 3d). However, the absolute numbers of Treg cells were normal due to a decrease in the patients’ CD3^+^ T, and CD4 + T cell absolute counts (Fig. 3d). Importantly, in vitro polarization of naive T cell into Treg lineage revealed that DIAPH1-deficient T cells could generate only a very small absolute number of regulatory T cells. However, the percentages of Treg cells were not dramatically low **(**Fig. 3e, f). These results suggest that conversion to regulatory T cells from naïve CD4^+^ T cells probably is affected in vitro more so than in vivo, and that the expansion of Treg cells is negatively affected by DIAPH1 deficiency.

**Fig. 4:**
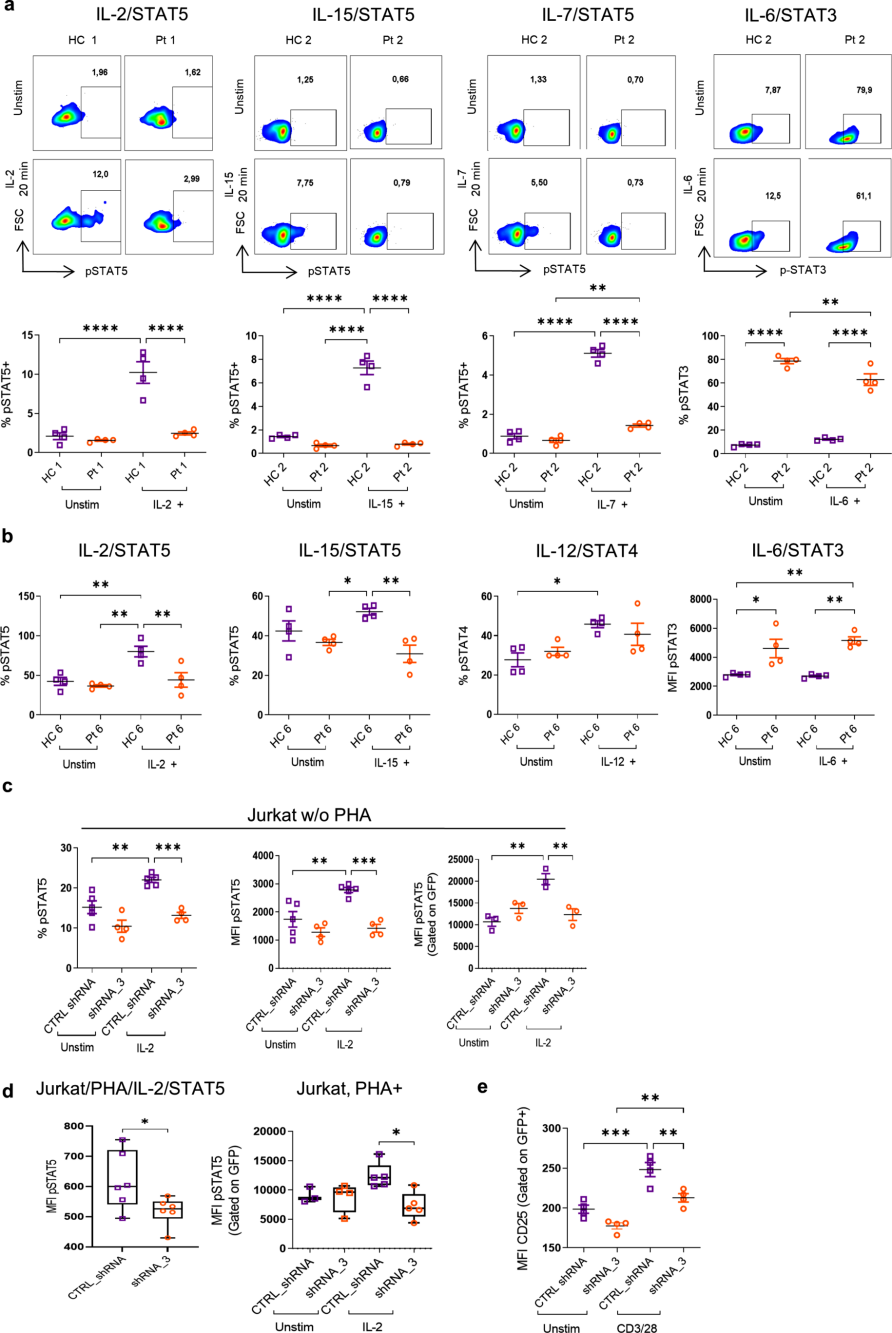
Impaired IL-2/IL-7/IL-15-dependent STAT5 phosphorylation in DIAPH1-deficient patient PBMCs and IL-2/STAT5 axis DIAPH1-silenced Jurkat T cells. **(a-b)** PBMCs from Pt1, 2, 6 and healthy control were rested for 2 h in the serum-free media, then stimulated with IL-2, IL-7, and IL-15 for p-STAT5, IL-6 for p-STAT3 and IL12 for pSTAT4 20 min. Then, fixed, permeabilized, and stained. **(a)** a representative flow chart and percentage and percentage bar chart for each condition for Pt1 and 2 were shown, four technical replicates run. **(b)** Percent phosphorylation chart for each condition was shown for Pt6, with 4 replicates. **(c)** After DIAPH1 silencing in Jurkat T cells for 48 h, cells were stimulated with IL-2 for p-STAT5 20 min at 37 °C. Stimulated cells fixed, permeabilized and stained for p-STAT5. Percentage and MFI bar chart are shown. **(d)** After DIAPH1 silencing in Jurkat T cells for 24 h, PHA was added for 24 h, cells were rested 2 h, and stimulated with IL-2 for p-STAT5 20 min at 37 °C. Stimulated cells fixed, permeabilized and stained for p-STAT5. Percentage and MFI bar chart are shown. **(e)** Forty-eight hours after DIAPH1 silencing in Jurkat T cells CD25 expression was quantified by MFI via surface staining and flow cytometry. For *p*-values *<0.05, **<0.01,***<0.001, ****< 0.0001

STAT5 plays a critical role in the expansion and survival of conventional T cells and Treg cells owing to its functions downstream of IL-2 receptor. To test whether DIAPH1-deficiency impacts STAT5 phosphorylation in response to IL-2 ligation, primary T cells from DIAPH1-deficient patients and healthy donors were activated with IL-2, and those experiments revealed a diminished IL-2-dependent STAT5 phosphorylation **(**Figs. 4a, b and S3b). Moreover, both IL-7 and IL-15-dependent STAT5 phosphorylation were markedly reduced **(**Fig. 4a, b). The defect in STAT5 phosphorylation was also observed in samples of Pt2 and Pt6 with the p.R351* and p.R322* mutations, respectively. However, we did not observe any significant reduction in STAT3 (perhaps even an elevation was observed) and STAT4 phosphorylation in response to IL-6 and IL-12, respectively **(**Figs. 4a, b and S3c).

To ensure that defective STAT5 phosphorylation is due to DIAPH1 but not an overlooked gene defect, we repeated the experiments using DIAPH1-silenced Jurkat T and primary T cells. Our findings confirmed a decrease in STAT5 phosphorylation when DIAPH1-silenced Jurkat cells with shRNA were activated with IL-2 **(**Fig. 4c). Due to low expression of CD25 by Jurkat cells, we activated them with PHA following transfection with shRNA in order to increase the surface expression of CD25 and rested them for 2 h in serum-free media. Similarly, STAT5 phosphorylation was also reduced in the DIAPH-silenced Jurkat cells compared with scrambled shRNA, suggesting DIAPH1-mediated regulation of the IL-2/STAT5 axis **(**Fig. 4d). Of note, CD25 surface expression was significantly lower on the cell surface of Jurkat cells after DIAPH1 knockdown, which may partly explain reduced STAT5 phosphorylation in response to IL-2 **(**Fig. 4e). However, we could not observe a significant difference in IL-2-mediated STAT5 phosphorylation in primary T cells which may be due to lower knockdown efficiency or compensatory mechanisms (Fig. S3d**)**. Collectively these results support a role for DIAPH1 in IL-2, IL-7 and IL-15-mediated STAT5 phosphorylation. The deficiency of these three signaling pathways may thus partly explain not only the Treg cell expansion, but also the non-Treg T cell activation and proliferation defects.

### The Proteomic Analyses of DIAPH1 Deficient CD4^+^ T Cells

**Fig. 5:**
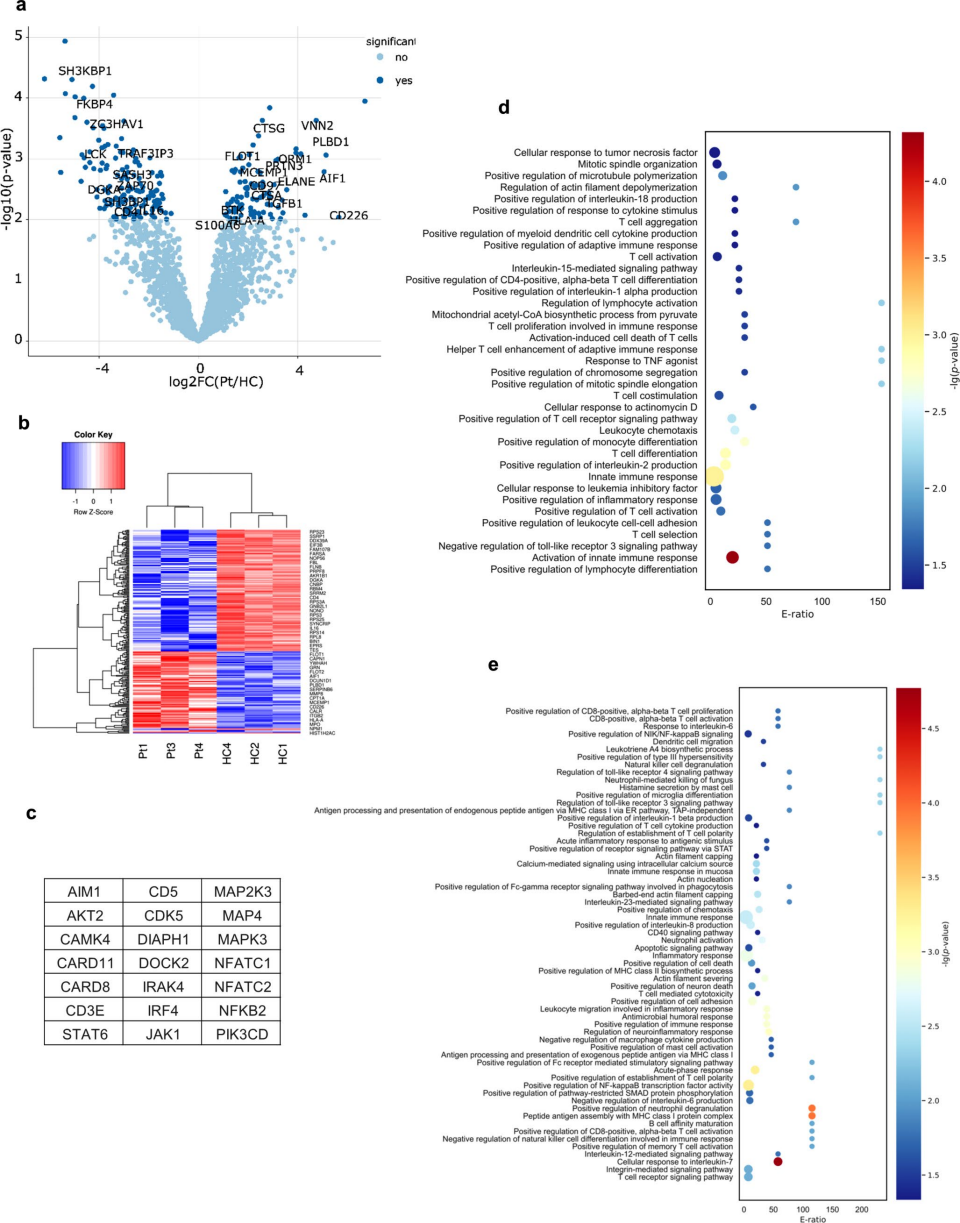
DIAPH1 deficiency alters intracellular proteome of anti-CD3/28 activated patient CD4 + T cells. **(a)** Volcano plot of differentially expressed proteins in DIAPH1-deficient patient CD4^+^ T cells (*P* < 0.01, log_2_ fold change (FC) > 1 or < -1) **(b)** Heatmap depicting the upregulated and downregulated proteins in DIAPH1-deficient patient CD4^+^ T cells. Due to size limitations, the names of specific proteins are listed directly in the figure. **(c)** select proteins which were not detected in DIAPH1 deficient CD4^+^ T cells (but present in those of the HC **(d)** Bubble chart of upregulated and **(e)** downregulated cellular processes in DIAPH1 deficient CD4^+^ T cells. 3 patients and 3 HC samples were used

To have a better understanding of T cell defects associated with *DIAPH1* deficiency, differential protein expression among 1694 proteins commonly expressed by both HCs and DIAPH1-deficient patients was analyzed using label-free quantitation. All the results, including a comprehensive list of protein identifications can be located in supplemental Table S3. After data processing and normalization, 233 differentially expressed proteins (DEPs) (*P* < 0.01, log_2_ fold change (FC) > 1 or < -1), including 91 upregulated DEPs and 142 downregulated DEPs, were identified (Table S4). Upregulated and downregulated proteins were visualized in the volcano plot and heat maps (Fig. 5a, b). Consistent with defects in T cell signaling, ZAP70, LCK, CD4, TRAF3IP3, SH3BP1, DGKA, and FKBP4 proteins were downregulated in DIAPH1-deficient CD4^+^ T cells. Importantly, the proteins unique to each group most likely represent missed protein assignments because of their low expression levels. Some proteins were below detection range in the patient samples which included DIAPH1, CD3e, CD5, and some MAP kinases consistent with reduced protein levels in DIAPH1 and impaired TCR signaling (Fig. 5c). Additionally, differentially regulated proteins were subjected to annotation based on Gene Ontology (GO) processes. In both upregulated (Fig. 5d) and downregulated **(**Fig. 5e), gene list enriched processes were visualized by bubble chart and revealed several immune processes including T cell activation, migration, differentiation, chemotaxis as well as microtubule and actin organization. Collectively, these results mark the critical roles played by DIAPH1 protein in CD4^+^ T cell biology and function and support the various abovementioned experimental evidence, which revealed major defects in T cells.

### DIAPH1 Deficiency Results in Numeric Reduction in Innate Lymphoid Cells and Functional Defects in Cytotoxic NK Cells

**Fig. 6:**
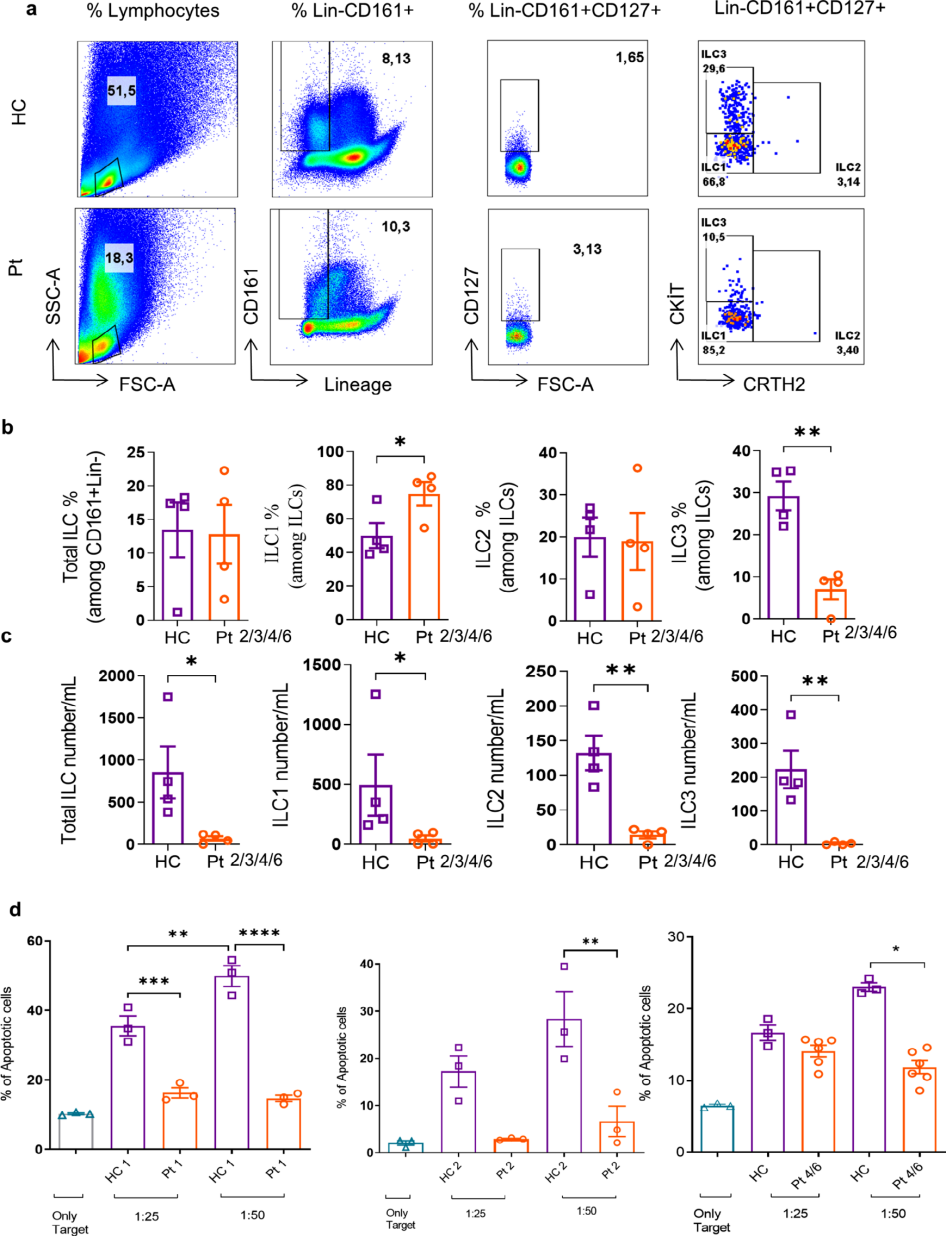
Reduced numbers of peripheral blood ILC subsets and impaired NK cell cytotoxic activity in DIAPH1-deficient patients. **(a)** Gating strategy for ILCs, representative plots for healthy controls (HC) and patients (Pt) PBMC. Total ILCs were gated as Lineage negative (TCRαβ^-^, TCRγδ^-^, CD34^-^, CD123^-^, CD94^-^, CD14^-^, BDCA2^-^, FcεRIα^-^, CD1a^-^, CD11c^-^, CD19^-^, B220^-^) CD3^-^CD161^+^ CD127^+^ Lin^-^ cells, ILC3s as cKit ^+^ CRTH2^-^ CD127 ^+^ CD161^+^ Lin^-^, ILC2s as CRTH2^+^ CD127^+^ CD161^+^ Lin^-^ and ILC1s as cKit^-^ CRTH2^-^ CD127^+^ CD161 ^+^ Lin^-^. **(b)** Percentage of total or subsets of ILCs in the peripheral blood of Pts and HCs. The samples from four Pts and four HCs were shown. **(c)** Absolute number of total or subsets of ILCs in the peripheral blood of Pts and HCs per mL of peripheral blood. **(d)** PBMCs from HCs and Pt1, 2, 4, and 6 were cocultured with labeled K562 cells as targets at 37 °C for 4 h. Target to effector ratio (T: E) was 1:25 or 1:50. The death in target cells stained with ANNEXIN V and 7-AAD was quantified. The error bars show +/- SEM. For *p*-values *<0.05, **<0.01,***<0.001, ****< 0.0001

Recently, we have reported numeric and functional defects in innate lymphoid cells in another actinopathy associated with DOCK8 deficiency [33]. Thus, we examined different subsets of helper ILCs (ILC1, ILC2, ILC3) and the cytotoxic NK activity in the PBMCs obtained from DIAPH1-deficient patients and healthy controls. The gating strategy for ILCs is given in Fig. 6a. All subsets of helper ILCs were dramatically reduced in the peripheral blood of all four patients tested **(**Fig. 6b, c). Both the frequency of ILC3 among all helper ILCs and their absolute numbers were reduced indicating a major negative impact of DIAPH1 deficiency on ILC3s (or ILC precursors). Additionally, helper ILC1 and ILC2 numbers are significantly reduced as well, consistent with a major reduction in the ILC3 quadrant which is also shown to contain precursors ILCs by others [34]. These findings argue that at least in some actinopathies, ILCs are negatively impacted underlining the importance of those genes in the maintenance/generation/function of these cells.

Lastly, we assessed the cytotoxic ability of PBMCs obtained from the Pt1, 2, 4, and 6 **(**Figs. 6d and S4a). Only Pt1 had low (compared to reference values (5–20%) NK cell percentage in PBMCs among six patients (Table S2). We used labeled-K562 cells as targets, and measured apoptosis on the target cells induced by equal number of patient derived PBMCs. These assays revealed reduced cytotoxic activity of DIAPH1-deficient PBMCs, suggesting a functional defect in NK cell cytotoxicity function. Since NK cells are not sorted, the reduction may also be attributed to a decrease in NK cell numbers in the peripheral blood. Therefore, we also measured NK cell surface activation markers CD69, KLRG1, NKp44, as well as granzyme B, IFN-γ and TNF-α among PBMCs cultured with or without K562 cells by gating on CD56^+^ or CD94^+^ NK cells (from Pt2) (Fig. S4b). These experiments revealed statistically significantly reduced CD69, NKp44 surface and Granzyme B and IFN-γ production by DIAPH1-deficient NK cells, collectively arguing for an impaired NK cell function in the absence of functional cellular DIAPH1. To definitively confirm that defects in NK cells are due to functional impairment not reduction in number, we sort purified NK cells via Miltenyi Microbeads and repeated the cytotoxicity experiments, which revealed reduced target killing (Fig. S5a). Importantly, impaired IL-2/STAT5 axis was also detectable in DIAPH1-deficient NK cells (Fig. S5b). In line with this, cultured pure NK cells (in the presence of IL-15), had slightly but significantly higher apoptosis, and reduced CD25 surface expression (Fig. S5c-e). Pt2’s NK cells were also biased in favor of CD56^high^ population, and perforin protein expression was reduced in both the CD56^high^ and CD56^int^ NK cells, whereas Granzyme B^+^ cells was only reduced in frequency in CD56^high^ subset (Fig. S5f-h). Altogether these data support the presence of qualitative and quantitative defects in helper ILCs and cytotoxic NK cells in human DIAPH1 deficiency.

## Discussion

Actinopathies arise due to mutations altering the function of genes involved in the formation, function and regulation of actin cytoskeleton. More than 20 monogenic inborn errors of immunity (IEI) diseases have been described in the literature to this date, each regulating distinct aspects of actin microfilament biology, elongation, activation, protrusion, branching, transcription etc. [35, 36]. Actinopathies manifest with immunological symptoms ranging from autoinflammation, autoimmunity, atopy to arthritis [35, 36]. DIAPH1, critical in actin filament nucleation/elongation, has first been associated with an IEI in 2021 by Kaustio et al. in a report which included 7 patients [4]. Our current work with 6 patients extends the cellular and molecular immunological defects of DIAPH1 deficiency and provides evidence for the critical roles played by DIAPH1 in T cell proximal signaling, STAT5-mediated signaling and Treg cell biology (Graphical Abstract). Importantly, our data provides the first evidence that DIAPH1 regulates helper ILCs and NK cell functions in humans.

DIAPH1 variants with various types of mutations have been described in the literature and were given in Table S5 [1]. Autosomal dominant mutations of DIAPH1, which are believed to be gain of function mutations, are associated with deafness with or without cytopenia (DFNA1). Several such heterozygous mutations have been described, some of which are in DID domain, others in the DAD domain all of which were believed to bypass the autoinhibitory regulation. Autosomal recessive loss of function mutations have been described in the literature, most of which are nonsense mutations (in the FH2 domain) or splice site variants (Table S5). Those mutations which have been studied functionally revealed drastic reduction in the protein levels [1]. The mutations described in the current study (p.R351* and p.R322* (as p.R331* for isoform 1) have been listed in the gnomAD, yet no functional validation studies have been provided to this day. The data presented herein confirm that the two described mutation result in dramatic loss of DIAPH1 protein and mRNA expression. Unlike the prior mutations, these two gain-of-stop mutations are in the FH3/DID domain.

Our results confirm that both variants result in major T cell-intrinsic proliferation and activation defects (with respect to CD69, CD25 expression) in T cells as shown with other LoF variants reported by Kaustio et al. [4]. This phenotype is also supported by the lymphopenia observed in 6 patients reported herein. *mDia1* KO mice were shown to have lymphopenia, activation and proliferation defects in response to anti-CD3/28 stimulation [20]. Percentage wise, CD8^+^ T cells were affected more severely than CD4^+^ T cells, although the absolute counts showed reduction for all T cells in mice [20]. Moreover, our findings revealed that DIAPH1 deficiency also impairs TCR signaling in human T cells, as evident in reduced ZAP70 and NF-κBp65 phosphorylation upon anti-CD3/CD28 ligation, as well as the proteomics data which showed reduced TCR signaling components (ZAP70, LCK, CD4, TRAF3IP3, SH3BP1, DGKA, FKBP4). TCR signaling in DIAPH1 deficiency has recently been assessed in mice by others [21], but not in humans to the best of our knowledge. Interestingly, this recent murine study suggested that *mDia1* KO CD8^+^ T cells did not have TCR signaling defect due to a possible compensatory role by mDia3, however, mDia1/mDia3 double KO CD8^+^ T cells showed impaired LAT, and downstream phosphorylation including SLP76, reduced proliferation and IL-2 production [21]. Unlike mDia KO murine CD8^+^ T cells, DIAPH1-deficient human T cells appear to be affected severely by the absence of DIAPH1. In addition, ZAP70 phosphorylation appears to be significantly affected by DIAPH1 deficiency, underlining a potential species-specific (or even CD4/CD8 specific) functional divergence. This contrasts with the deficiency of other actinopathy-associated proteins such as WASP [37], CARMIL2 [38] for which normal ZAP70 activation was reported, however TCR signaling beyond ZAP70 has been shown to be impaired in those deficiencies. In Arp2/3 defective mice, which results in an actin branching defect, TCR surface expression has been shown to be reduced [39]. Therefore, it is conceivable that reduced ZAP70 phosphorylation in DIAPH1 deficient T cells in the current study may, in part, be due to reduced TCR surface expression. Indeed, comparison of CD3 MFI between two patients and healthy controls revealed reduced surface expression of CD3 (in addition to reduced CD4/CD3/IRF4/NFATc expression from proteomics data). Thus, these data may explain reduced TCR activation in DIAPH1-deficient human T cells.

Our results also revealed major cytokine signaling defects especially associated with common gamma chain (γc)-dependent cytokines including IL-2, IL-7, IL-15 and associated STAT5 phosphorylation, but not that of STAT3, nor STAT4. These results are reminiscent of other actinopathies [24, 25, 29, 33, 40, 41]. Prior research have revealed impaired STAT5 phosphorylation and nuclear translocation in response to IL-2, impaired STAT3 phosphorylation in response to IL-6, and IL-23 in DOCK8 deficiency in different immune cell types [24, 25, 29, 33, 40, 41]. Importantly coimmunoprecipitation studies revealed an interaction between DOCK8 and STAT3 [40]. A similar defect in IL-2/STAT5 axis was reported in T cells of Wiskott-Aldrich syndrome (WAS) interacting protein (WIP)-and WASP double knockout mice [42]. A physical interaction (direct or indirect) between DIAPH1 and STAT5 is possible but needs to be further studied in detail. Such an interaction may be indirect and may be in the form of support by cytoskeleton mediated by DIAPH1-mediated actin nucleation activity as well. Alternatively, impaired phosphorylation of STAT5 in response to IL-2, IL-7, IL-15 could also be due to reduced surface expression of receptor components, or downstream regulators of STAT5. Our data indicate that reduced surface expression of IL-2Rα (CD25) may partly explain impaired IL-2/STAT5 signaling in both T and NK cells. Proteomics data also revealed reduced JAK1, STAT6 levels in DIAPH1-deficient patient CD4^+^ T cells. These data also underline that different cytoskeleton components may preferentially regulate activity of select STATs but not all.

Actinopathies, including DOCK8, WASP and CDC42 deficiencies, are associated with migration defects of various immune cells [43]. Migration defects have been described for various immune cell subsets in murine mDia1 deficiency. mDia1 deficient T and dendritic cells have been reported to have impaired migration in vitro or in vivo [20, 44]. Other cells or cell lines have been also studied and were shown to have migration defects in DIAPH1 deficiency [45]. Our study provides ex vivo evidence from DIAPH1-deficient human PBMCs and confirms those earlier findings with murine immune cells and human mast cells.

In addition, DIAPH1 deficient PBMCs appear to have a survival disadvantage over healthy PBMCs in culture conditions, as evident in apoptosis staining. Some actinopathies have been studied in this regard and DOCK8 (perhaps more) deficiency has been reported to lead to more T cell death [46]. Thus, our results underline the common theme shared by actinopathies. The reduced IL-7, IL-15 and IL-2 mediated signaling pathways may partly explain these results since those cytokine-signaling pathways also regulate the survival of T and NK cells.

Our results revealed for the first time that human DIAPH1 deficiency is associated with major ILC defects. ILC1 subset includes both cytotoxic NK cells and helper ILCs. NK cell percentages and numbers were not similarly reduced across all six DIAPH1-deficient patients. However, cytotoxicity assays on PBMC and sorted primary NK cells revealed impaired cytotoxicity and cytokine production for DIAPH1-deficient NK cells. NK cell function defects have been described for other actinopathies [36], including DOCK8 [47], WASP [48], WIP [49], Arp2/3 [50] and perhaps CARMIL2 [51, 52]. Detailed studies previously have shown that DIAPH1 overlayed with the MTOC and microtubules was observed surrounding the centrosome, additionally, DIAPH1 was overlayed with Arp2/3-independent filopodia in both Jurkat and human CD4^+^ T cells [50]. More detailed investigation of the NK-target cell immunological synapse will reveal the molecular mechanisms of the defects associated with DIAPH1 deficiency. In addition to cytotoxicity defects, NK cells appear to be deficient in perforin production, and also IL-2/STAT5 axis, which further impair their function. Additionally, our results show that peripheral helper ILC subsets rely on DIAPH1 for their maintenance or generation. It is unclear whether this numeric reduction in the peripheral blood in ILC1, 2 and 3 is applicable to tissues. Our prior studies with DOCK8-deficient mice revealed that indeed in tissues, especially the gut, ILCs require cytoskeletal regulator DOCK8 for expansion and function [41] which was corroborated in DOCK8-deficient patients [33]. Given that IL-7/STAT5 IL-15/STAT5 and IL-2/STAT5 signaling modules are impaired in DIAPH1 deficient T cells argue that these signaling defects would also negatively impact ILCs subsets as they are critical in ILC subsets’ survival and expansion.

Our study has limitations as well. Potential interactions of DIAPH1 with various STAT molecules need to be investigated further. Additionally, characterization of T cells and ILCs in the human tissues will further our understanding of DIAPH1 mediated regulations beyond the peripheral blood. Lastly, due to the abundance of experiments, and distribution of the patients across a wide region some experiments could be performed with some of the patients. Nevertheless, in summary, the data presented in this work presents a detailed picture of cellular and molecular immunological defects in DIAPH1-deficient human T, NK cells and helper ILC subsets thus far, expanding the cellular and immunological manifestations of DIAPH1 deficiency in humans, and contributing to our understanding of the mechanisms behind why DIAPH1-deficient patients are prone to infections and cancer [53].

## Electronic Supplementary Material

Below is the link to the electronic supplementary material.

Supplementary Material 1

Supplementary Material 2

Supplementary Material 3

Supplementary Material 4

Supplementary Material 5

Supplementary Material 6

Supplementary Material 7

Supplementary Material 8

Supplementary Material 9

Supplementary Material 10

Supplementary Material 11

Supplementary Material 12

## Data Availability

Proteomics data is available on request.

## Abbreviations

DIAPH1: Diaphanous related formin 1
DRF: Diaphanous-related formin
IL-2: Interleukin 2
STAT: Signal transducer and activator of transcription
NK: Natural killer
ILC: Innate lymphoid cell
WES: Whole exome sequencing
PBMC: Peripheral blood mononuclear cell
DAD: Diaphanous activatory domain
DID: Diaphanous inhibitory domain
FH: Formin Homology
CC: Coiled coiled
CD: Cluster of differentiation
qPCR: Quantitative polymerase chain reaction
