# Correction to: DIAPH1-Deficiency is Associated with Major T, NK and ILC Defects in Humans

**DOI:** 10.1007/s10875-024-01832-4

**Published:** 2024-11-16

**Authors:** Zehra Busra Azizoglu, Royala Babayeva, Zehra Sule Haskologlu, Mustafa Burak Acar, Serife Ayaz-Guner, Fatma Zehra Okus, Mohammad Bilal Alsavaf, Salim Can, Kemal Erdem Basaran, Mehmed Fatih Canatan, Alper Ozcan, Hasret Erkmen, Can Berk Leblebici, Ebru Yilmaz, Musa Karakukcu, Mehmet Kose, Ozlem Canoz, Ahmet Özen, Elif Karakoc-Aydiner, Serdar Ceylaner, Gülsüm Gümüş, Huseyin Per, Hakan Gumus, Halit Canatan, Servet Ozcan, Figen Dogu, Aydan Ikinciogullari, Ekrem Unal, Safa Baris, Ahmet Eken

**Affiliations:** 1 Department of Medical Biology, Faculty of Medicine, Erciyes University, Kayseri, 38039 Türkiye; 2 Genome and Stem Cell Center, Kayseri, 38039 Türkiye; 3 Division of Pediatric Allergy and Immunology, Department of Pediatrics, Faculty of Medicine, The Istanbul Jeffrey Modell Diagnostic Center for Primary Immunodeficiency Diseases, The Isil Berat Barlan Center for Translational Medicine, Marmara University, Istanbul, Türkiye; 4 Division of Pediatric Allergy and Immunology, Department of Pediatrics, Faculty of Medicine, Ankara University, Ankara, Türkiye; 5 Department of Molecular Biology and Genetics, Izmir Institute of Technology, Izmir, Türkiye; 6 Erciyes University Medical School, Kayseri, Türkiye; 7 Department of Physiology, Faculty of Medicine, Erciyes University, Kayseri, 38039 Turkey; 8 Division of Pediatric Hematology and Oncology, Department of Pediatrics, Faculty of Medicine, Erciyes University, Kayseri, 38039 Turkey; 9 Department of Medical Genetics, Faculty of Medicine, Ankara University, Ankara, Türkiye; 10 Division of Pediatric Pulmonology, Department of Pediatrics, Faculty of Medicine, Erciyes University, Kayseri, 38039 Türkiye; 11 Department of Pathology, Faculty of Medicine, Erciyes University, Kayseri, 38039 Türkiye; 12 Intergen, Medical Genetics, Ankara, Türkiye; 13 Division of Pediatric Radiology, Department of Radiology, Faculty of Medicine, Erciyes University, Kayseri, Türkiye; 14 Division of Pediatric Neurology, Department of Pediatrics, Faculty of Medicine, Erciyes University, Kayseri, 38039 Türkiye; 15 Department of Biology, Faculty of Science, Erciyes University, Kayseri, 38039 Türkiye; 16 Medical Point Hospital, Hasan Kalyoncu University, Gaziantep, Türkiye; 17 Medical Point Hospital, Pediatric Hematology Oncology and BMT Unit, Gaziantep, Türkiye

**Correction to: Journal of Clinical Immunology (2024) Aug 9;44(8):175**

**https://doi.org/10.1007/s10875-024-01777-8**

In this article, Ekrem Unal, Safa Baris, Ahmet Eken should have been denoted as co-corresponding authors.

The original version has been corrected.

