# Supplementary material for: DIAPH1-Deficiency is Associated with Major T, NK and ILC Defects in Humans
Source: J Clin Immunol. 2024 Aug 9;44(8):175. doi: 10.1007/s10875-024-01777-8 (PMC11315734; doi:10.1007/s10875-024-01777-8)
Supplement: Supplementary file 1 — Supplementary Material 1 [file 10875_2024_1777_MOESM1_ESM.docx]

**Supplementary Methods**

**Isolation, Culture and Surface /Intracellular Staining of Cells**

According to the manufacturer's protocol, peripheral blood mononuclear cells (PBMCs) were isolated from the blood via density gradient using Ficoll-Paque Plus (Cat#GE17-1440-03). PBMCs were counted with a hemocytometer using Trypan Blue (Cat# SKU: 03-102-1B) and used for surface/intracellular staining (ICS), proliferation, cytotoxicity, phosphorylation, migration, and Treg differentiation assays. The cells for surface staining were Fc-blocked with Human TruStain FcX ™ for 5 min and then the cells were treated with Alexa flour 700-CD3 (clone: PIT3A), FITC-CD4 (clone: A161A1), PerCP/Cy5.5-CD127, PerCP/Cy5.5-CD25, PE-CD69 combination for 30 min at 4 °C in staining Buffer (PBS supplemented with 2% FBS).

For ICS, PBMCs were resuspended in complete RPMI-1640 medium (BI, Cat# 01-106-1A) ( (including 10% FBS and essential (Gibco, cat# 11130051) and non-essential amino acids (Gibco, cat# 11140050), Anti-Anti (Antibiotic-Antimycotic, Gibco) and stimulated with anti-human CD3 (clone: OKT3) (1µg/mL) and anti-human CD28 (Clone: CD28.2) (1 μg/mL) overnight into 96-well round-bottom plates. Then cells were stimulated with Phorbol-Myristate-Acetate (PMA, Sigma, P1585-1MG) (50ng/mL), Ionomycin (Sigma, Cat#I3909) (1µg/mL), Golgi Plug (BD, cat# 555029) (1 µl/mL) for 4 hours at 37°C. The cells were fixed and permeabilized using BD Cytofix/Cytoperm™ Plus intracellular staining kit. APC IL-17 (clone: BL168), PercpCy5.5 IL-22 (clone: 2G12A41), APC IL-2 (clone: MQ1-17H12), BV421 IFNg (clone: B27), PercpCy5.5 TNFa (clone: Mab11) combination was used for ICS. The cells were run on FACSAria III, and data analysis was performed using FlowJo software.

The True-Nuclear ™ Transcription Factor Buffer Set (Biolegend, cat# 424401) was used to stain Treg cells for FOXP3. For each sample, 90 µl of fresh Transcription Factor Fixation Buffer (1X) was used to fix cells for 30 min at room temperature; the pellet was obtained by centrifugation at 400g for 3 minutes. Each sample was permeabilized by the addition of 150 µl perm buffer, and washing twice. Finally, the samples were stained with anti-human PE- FOXP3 (clone: 150D) for 30 min, and cells were washed. Prior to ICS cells were stained for CD3, CD4, and CD127. Stained cells were run on FACSAria III, and data analysis was performed using FlowJo software (BD Biosciences).

ILCs were gated as follows based on our prior publications[1]: Total ILCs were gated as Lineage negative (TCRαβ-, TCRγδ-, CD34-, CD123-, CD94-, CD14-, BDCA2-, FcεRIα-, CD1a-, CD11c-, CD19-, B220-) CD3-CD161+CD127+ Lin- cells, ILC3s as cKit+CRTH2- CD127+CD161+Lin-, ILC2s as CRTH2+CD127+CD161+ Lin- and ILC1s as cKit- CRTH2- CD127+CD161+Lin-.

**DIAPH1 Staining and Analyses**

The healthy control and the patient's PBMCs were centrifuged at 400g for 3 minutes. The cells were fixed and permeabilized using BD Cytofix/Cytoperm™ Plus ICS kit. 5µl Fc-block and 10 µl FBS were added to the cells and incubated for 5 minutes. Polyclonal rabbit anti-human DIAPH1 primary antibody was added (1:100 dilution) with perm buffer and kept at +4ºC overnight. Only perm buffer was added to the negative control. After treatment cells were washed twice and 10 µl FBS was added to the cells. Then all cells were stained with secondary antibody (Anti-rabbit IgG-FITC) (1:100 dilution). The cells were incubated for 1 hour at +4°C and washed by centrifugation at 400g for 3 minutes with staining buffer (PBS with 2% FBS). The cells were analyzed in Flow cytometry. For confocal microscopy, cells were spun, supernatant removed and resuspended in leftover staining buffer. 15 µl of the cells were mounted on a glass slide along with mounting medium containing DAPI (4 ′, 6-diamidino-2-phenylindole, Thermo Scientific, USA). The slides were covered with a cover slip, sealed and left for overnight at +4ºC, and analyzed under a confocal microscope (LSM 900 Airyscan 2, Axio Scop, ZEISS, Germany).

**T Cell Proliferation**

The isolated PBMCs were stained with Tag-it-violet (Biolegend # 425101) as described by the manufacturer. Labeled cells were cultured with phytohemagglutinin (PHA, 1µg/mL) and soluble anti-human CD3 (1µg/mL) and anti-human CD28 (1µg/mL) for 4 days. Following a wash step once with staining buffer, the cells were run on FACSAria III.

**Apoptosis Staining**

PBMCs (10^5^ cells/well) were stimulated with soluble anti-CD3/anti-CD28 (1 µg/mL) in 96-well plates overnight and stained with 7-AAD using BioLegend's FITC Annexin V Apoptosis Detection Kit and analyzed on FACSAria III.

**Cytotoxicity Assay**

K562 cells were stained with Tag-it-violet (Biolegend # 425101). Then the isolated-PBMCs from healthy donors and patients were cocultured with labeled K562 cells (20,000/well) at 37°C for 4 hours at a 1:25 or 1:50 ratio (K562: PBMC). Cells were stained with 7-AAD using BioLegend's FITC Annexin V Apoptosis Detection Kit according to the manufacturer's protocol, and then labeled K562 cells were gated and analyzed for apoptosis markers on FACSAria III.

**Phospho-Flow**

PBMCs were stimulated with phytohemagglutinin (PHA) (1 μg/mL) overnight in complete RPMI medium at 37°C overnight. The cells were washed twice in serum-free complete RPMI medium, resuspended in serum-free complete RPMI medium, distributed into 96-well round bottom plates 10-50 x10^4^ /well density and rested for 2 hours. Then, cells were stimulated in 100μL of complete medium with IL-2 (500 Units / mL) for 20 min at 37°C for IL-2 mediated STAT5 phosphorylation. The rested cells were also stimulated with IL-23 (50 ng/mL) and IL-6 (100 ng/mL) for p-STAT3, IL-12 (20 ng/mL) for p-STAT4, IL-7(20 ng/mL) and IL-15 (10 ng/mL) for p-STAT5 individually 20 min at 37°C. The stimulations were terminated by fixing with the same volume of 4% PFA for 15 minutes at 37°C. Cells were washed twice with PBS and permeabilized at -20ºC with 150 µl cold methanol/well for 30 min. After washing with staining buffer, the cells were separately stained with PE-pSTAT3 (Clone: 13A31), PE-pSTAT4 (Clone:4LURPIE), PE p-STAT5 (Clone: Y694).

After DIAPH1 silencing, Jurkat cells were kept on ice in serum-free RPMI medium for 1 hour. Then, cells were mixed with primary anti-CD3/28 for 30 min in serum free medium. At the end of the period, the secondary polyclonal antibody anti mouse IgG (1mg/mL) 20ug/mL was added to the cells and incubated for 30 minutes at 37ºC. The cells were washed once with PBS. It was pelleted at 400g for 5 minutes and the supernatant was discarded. Stimulation was terminated by fixing with the same volume of 4% PFA for 15 minutes at 37°C. Cells were washed twice with PBS and permeabilized at -20ºC with 150 µl cold methanol/well for 30 min. After washing with staining buffer, the cells were separately stained with PERCP-Cy5.5 p-ZAP70, PE-Cy7 p-ERK, PE Dazzle584 p-NF-κBp65 antibodies for 30 min and analyzed on FACS Aria III.

**Migration Assay**

PBMCs were rested in serum-free RPMI medium for 4 h at 37◦C. Then, 300 μL of cells in serum-free RPMI medium were taken and loaded into 5 μm porous polycarbonate inserts (Corning) (1 ×10^4^ to cells per insert). The bottom wells include 500 μL complete RPMI medium (%10 FBS). A serum free RPMI medium was -used for the control group. After 4 hours of incubation at 37◦C, -migrated cell count was measured by Flow cytometry using Spherotech counting beads (Cat#ACBP-20-10) as described previously[1].

**Treg Differentiation Assays**

CD4+ naïve T cells were sorted from PBMCs by naïve CD4+ T cell isolation kit II, human (Miltenyi 130-094-131). Cells were counted under a hemocytometer and were cultured in 96-well plates with complete RPMI-1640 medium containing coated anti-human CD3 (2 µg/mL), soluble anti-human CD28 (1µl/mL), human IL-2 (50 ng/mL), and human TGF-β (5ng/mL) at 37◦C for 3 days. On the 3rd day, the complete RPMI medium containing soluble anti-human CD28, human IL-2, and human TGF- β of the cells was refreshed. After 2 days of culture at 37ºC, FOXP3 + cells were stained with surface and intracellular staining. FOXP3+ cells were quantified by Flow cytometry as described previously[2].

**Real-time qPCR**

Cells were collected in 300 µl Qiazol (cat# 79306). Then total RNA was extracted by phenol chloroform extraction. cDNA was synthesized using the iScript cDNA synthesis Kit (cat# 1708891). LightCycler® 480 (Roche) Instrument and SYBR Green (Bio-Rad, cat# 1725124) method was used to detect PCR products. Relative gene expression was calculated by the ΔΔCT method. Expression was normalized over 18S ribosomal RNA message. Primer sequences are given in **Table S6**

**Enzyme-Linked Immunosorbent Assay (ELISA)**

The plasma of blood samples was taken for ELISA and stored at -80ºC until use. The 96-well plates were coated with coating buffer overnight. Biolegend Human IL-17A ELISA- MAX, Human IL-22 ELISA-MAX, and Human GMCSF ELISA-MAX kits were used to run ELISA. The manufacturer’s protocol was followed. TNF and IL-4 levels were measured using multiplex ELISA (LEGENDPlex, cat# 741028).

**Knockdown of *DIAPH1***

shRNA in a retroviral construct for the knockdown of *DIAPH* was purchased from Origene (cat# TG313475). The plasmid construct was transfected to the Jurkat T cell line using the Neon® Transfection System (Invitrogen, USA) kit. The cells were centrifuged at 400 g for 5 minutes, washed with PBS (without Ca^+2^ and Mg^+2^) and centrifuged at 400 g at room temperature for 5 minutes. Cells were resuspended at 1 x 10^7^/mL in R buffer (for cell line), and 100 μL of cells were taken and transfected with 200 ng *DIAPH1* shRNA. Transfection was performed at Voltage 1350, Pulse Width 10, Pulse Number 3. After transfection, cells were seeded in 2 mL of antibiotic-free medium in 6 wells. After the cells were cultured in this medium for 2 hours, antibiotics were added and incubated at 37◦C for 2 days. Transfected cells showed reduced expression of theDIAPH1. At the end of the period, the knockdown was verified with real-time q-PCR and/or flow cytometry. Proliferation, signaling and IL-2 production were assessed in transfected cells.

**Proteomics Analyses**

CD4+ T cells were sorted from PBMCs isolated from both healthy controls and patients (Pt 1, 5, 6) using FACS AriaIII sorter following staining with anti-CD4 and CD3 antibodies. The cells were activated with soluble anti-CD3 and anti-CD28 (1µg/mL each) for 4 hours. Then the cells were washed with PBS twice and the pellet was frozen for the proteomics analyses. The pellet was thawed, and processed with the InStage Tip digestion method as described previously by Kulak and colleagues [3]. One million cells per sample pellet was dissolved in 100 μL Lysis Buffer (6 M Guanidinium chloride, 40 mM CAA, 10 mM TCEP, 25 mM Tris-HCl pH:8,5). The lysates were first boiled for 5 minutes, then sonicated in an ice-filled ultrasonic water bath for 5 min then centrifuged at 20,000 g for 15 min. The supernatants containing protein were collected and processed further. Twenty μL of each supernatant was mixed with 40 μL dilution buffer (25 mM Tris-HCl pH 8.5, % 10 ACN) containing 240 ng Lys-C (Promega, WI, USA), and run through InStage tips that were previously prepared by using 3 SDB-RPS extraction disks (3M Empore, MN, USA) overnight at 37°C. Subsequently, 800 ng Trypsin-Gold (Promega, WI, USA) was added to the Stage tips, mixed well, and incubated for 4 h. Then, 140 μL loading buffer (1% TFA) was added to each tip, and centrifuged at 2,000 g. The peptide-loaded disks were then washed 4 times with a 100 μL washing buffer. Based on their hydrophobic properties, the peptides were eluted from the disks in three fractions by using 60 μL of each of the following elution buffers: SDB-RPS2 (100 mM Ammonium formate, 55% ACN, 0.5% Formic Acid), SDB-RPS1 (100 mM Ammonium formate, 35% ACN, 0.5% Formic Acid) and Buffer X (80% ACN, 0.125% Ammonia). The elutions were lyophilized with SpeedVac and stored at −20°C until the LC-MS/MS analysis.

To conduct LC-MS/MS, we utilized a Dionex UltiMate 3000 RSLCnano UHPLC for sample loading onto a Pepmap100 C18 5um trap column (0.3x5mm, Thermo Scientific). Subsequently, peptides were separated using an Easy Spray analytical column (PepMap RSLC C18; 75 µm × 25 cm; Thermo Scientific). Peptide separation involved a linear gradient of 2–35% ACN/0.1% FA concentration, with a flow rate of 300 nL/min over 115 minutes. Analysis was performed using an Orbitrap Fusion Tribrid mass spectrometer (Thermo Scientific) employing a data-dependent acquisition strategy with the following parameters: Full ion scan mode recorded all MS spectra (MS1) profiles from 375– 1500 m/z in the Orbitrap at 120,000 resolution. Precursor ions were selected using top speed mode at a cycle time of 3  s to the ions over intensity threshold 1,0e4 with dynamic exclusion of 45 s. Normalized collision energy was set to 35 (high energy collisional dissociation (HCD)), and fragments were analyzed in the ion trap.

The RAW data were processed using MaxQuant [4] with default settings. MSMS spectra was searched against UniProt human database (UP000005640) Trypsin/P was specified as the enzyme with a maximum of two allowed missed cleavages. Fixed modifications included cysteine carbamidomethylation, while variable modifications will contain methionine oxidation and protein N‐terminal acetylation. A global false discovery rate of 1% was implemented for both proteins and peptides. The match‐between‐runs and re‐quantify options were enabled. We set the minimum number of MSMS counts to 3 and the minimum razor peptides to 2 for selecting valid identifications.

For comparing protein abundances between healthy control samples (n=3) (HC) and patient (Pt) samples (n=3), calculated using the label-free quantitation score (MaxLFQ) from MaqQuant software, we utilized a modified version of the limma R package known as DEqMS. To enhance comparability, we applied variance stabilization normalization (Vsn) to Quantitation values [5] Missing values were deliberately not imputed for a more stringent evaluation of quantitative proteomics.To visually represent our findings, we employed the ggplot2 and ggplot R packages to generate volcano plots and heatmap visualizations highlighting differentially expressed proteins.

Gene Ontology Biological Process analysis of identified proteins was conducted via StringDB[6] and Heatmap Illustrator (HemI 2.0)[7]. GO-BP enrichment was performed using default parameters of tools and results were represented as a bubble chart.

**References**

1. Eken A, Yetkin MF, Vural A, Okus FZ, Erdem S, Azizoglu ZB, et al. Fingolimod alters tissue distribution and cytokine production of human and murine innate lymphoid cells. Front Immunol. 2019;10:1–12.

2. Eken A, Cansever M, Somekh I, Mizoguchi Y, Zietara N, Okus FZ, et al. Genetic Deficiency and Biochemical Inhibition of ITK Affect Human Th17, Treg, and Innate Lymphoid Cells. J Clin Immunol. Journal of Clinical Immunology; 2019;39:391–400.

3. Kulak NA, Pichler G, Paron I, Nagaraj N, Mann M. Minimal, encapsulated proteomic-sample processing applied to copy-number estimation in eukaryotic cells. Nat Methods. 2014;11:319–24.

4. Cox J, Mann M. MaxQuant enables high peptide identification rates, individualized p.p.b.-range mass accuracies and proteome-wide protein quantification. Nat Biotechnol. 2008;26:1367–72.

5. Välikangas T, Suomi T, Elo LL. A systematic evaluation of normalization methods in quantitative label-free proteomics. Brief Bioinform. 2018;19:1–11.

6. Szklarczyk D, Kirsch R, Koutrouli M, Nastou K, Mehryary F, Hachilif R, et al. The STRING database in 2023: protein-protein association networks and functional enrichment analyses for any sequenced genome of interest. Nucleic Acids Res. Oxford University Press; 2023;51:D638–46.

7. Ning W, Wei Y, Gao L, Han C, Gou Y, Fu S, et al. HemI 2.0: an online service for heatmap illustration. Nucleic Acids Res. Oxford University Press; 2022;50:W405–11.
