# Supplementary material for: DIAPH1-Deficiency is Associated with Major T, NK and ILC Defects in Humans
Source: J Clin Immunol. 2024 Aug 9;44(8):175. doi: 10.1007/s10875-024-01777-8 (PMC11315734; doi:10.1007/s10875-024-01777-8)
Supplement: Supplementary file 2 — Supplementary Material 2 [file 10875_2024_1777_MOESM2_ESM.docx]

| **Characteristics** | **Patients** | | | | | |
| --- | --- | --- | --- | --- | --- | --- |
|  | **F1/ P1-Erciyes** | **F2/ P2- Erciyes** | **F3/ P3- Marmara** | **F3/P4-Marmara** | **F4/P5-Marmara** | **F5/P6-Ankara** |
| **Current Age** | **11** | **10** | **16** | **11** | **2** | **6** |
| **Gender** | **F** | **F** | **F** | **M** | **F** | **F** |
| **Mutation** | **NM_005219.4**  **c.1051C>T,**  **p. Arg351*** | **NM_005219.4**  **c.1051C>T,**  **p. Arg351*** | **NM_005219.4**  **c.1051C>T,**  **p. Arg351*** | **NM_005219.4**  **c.1051C>T,**  **p. Arg351*** | **NM_005219.4**  **c.1051C>T,**  **p. Arg351*** | **NM_001079812.3**  **c.964C>T,**  **p. Arg322*** |
| **Consanguineous** | **+** | **+** | **+** | **+** | **+** | **+** |
| **Symptom Age of onset (months)** | **7** | **10** | **6** | **6** | **4** | **2** |
| **Microcephaly** | **+** | **+** | **+** | **+** | **+** | **+** |
| **Failure to thrive** | **+** | **+** | **+** | **+** | **+** | **+** |
| **Mental Retardation** | **+** | **+** | **+** | **+** | **+** | **+** |
| **Epilepsy** | **+** | **+** | **+** | **+** | **+** | **+** |
| **Defect of vision** | **+** | **+** | **-** | **-** | **+** | **+** |
| **Recurring Infections** | **+** | **+** | **+** | **+** | **+** | **+** |
| **Severe Infection** | **+** | **+** | **+** | **+** | **-** | **+** |
| **Types of Infection** | **Recurrent acute**  **otitis media, Recurrent Pneumonia, bronchiolitis, sinusitis, EBV viremia, Clostridium difficile** | **Recurrent acute**  **otitis media, Pneumonia, sinusitis,** | **Pneumonia,**  **sinusitis,**  **paronychia** | **Pneumonia, sinusitis,**  **otitis, orofacial**  **herpes, sepsis** | **Moniliasis** | **Recurrent acute**  **otitis media,**  **Mastoiditis,**  **EBV viremia,**  **Viral respiratory**  **tract infections**  **(Rhinovirus)**  **Recurrent urinary**  **tract infections**  **(E. Coli)**  **Long Covid-19**  **infection** |
| **Other** | **Primary pulmonary parenchymal refractory plasmablastic lymphoma, Autoimmune hemolytic anemia,** **Immune thrombocytopenia** | **Autoimmune hemolytic anemia, Immune thrombocytopenia** | **Not reported** | **Autoimmune hemolytic anemia** | **Autoimmune hemolytic anemia** | **Autoimmune**  **hemolytic**  **anemia,**  **Mediastinal and**  **abdominal**  **lympadenopathy**  **Hepatomegaly** |
| **Treatment** | **Chemotherapy including rituximab, doxorubicin, cyclophosphamide, prednisone,**  **IVIG, TMP/SMX, Fluconazole, (Lymphoma treatment)** | **IVIG, TMP/SMX, Fluconazole** | **IVIG, TMP/SMX, Fluconazole,** | **IVIG, TMP/SMX, Fluconazole, levetiracetam** | **IVIG, TMP/SMX, Fluconazole, Vigabatrin, levetiracetam** | **IVIG, TMP/SMX, Fluconazole, Rituximab,**  **Levotirasetam,**  **Phenobarbital**  **Ursodeoxycholic acid** |
| **Last status** | **Deceased** | **Alive** | **Alive** | **Deceased** | **Alive** | **Deceased** |

**TABLE S1. Demographic and clinical characteristics of the patients with *DIAPH1* mutation**

Abbreviations: EBV- Epstein-Barr virus, IVIG- intravenous immunoglobulin, TMP/SMX- trimethoprim sulfamethoxazole, F-female, M-male
