# Supplementary material for: DIAPH1-Deficiency is Associated with Major T, NK and ILC Defects in Humans
Source: J Clin Immunol. 2024 Aug 9;44(8):175. doi: 10.1007/s10875-024-01777-8 (PMC11315734; doi:10.1007/s10875-024-01777-8)
Supplement: Supplementary file 3 — Supplementary Material 3 [file 10875_2024_1777_MOESM3_ESM.docx]

**TABLE S2.** Immunological evaluation of the *DIAPH1* mutation patients

| Parameters | F1/ P1-  Erciyes | F2/ P2- Erciyes | F3/ P3- Marmara | F3/P4-Marmara | F4/P5-Marmara | F5/P6-Ankara |
| --- | --- | --- | --- | --- | --- | --- |
| Leukocyte count (/mm^3^) | 5200 | 6090 | 7000 | 4800 | 8230 | 10230 |
| Absolute lymphocyte count (/mm^3^) | 1010 | 910 | 700 | 300 | 2890 | 1620 |
| Absolute neutrophil count (/mm^3^) | 3740 | 4390 | 4800 | 2600 | 4000 | 7430 |
| Absolute monocyte count (/mm^3^) | 310 | 500 | 600 | 300 | 1140 | 670 |
| Absolute eosinophil count (/mm^3^) | 140 | 260 | 780 | 1560 | 110 | 410 |
| IgG (mg/dl) | 1241 | 1100 | 748 | 873 | 1072 | 606* (745-1804) |
| IgA (mg/dl) | 185 | 446 | 28 | 26 | 63 | 648 (57-282) |
| IgM (mg/dl) | 56.8 | 43 | 89 | 84 | 79 | 33(78-261) |
| IgE (IU/ml) | 367 | 18 | 54 | 2.9 | 1.42 | <2 (0-150) |
| Specific antibody titers |  |  |  |  |  |  |
| Anti Hbs IgG (mIU/ml) | neg | neg | neg | neg | pos | neg |
| Anti-measles IgG (IU/l) | neg | neg | neg | neg | neg | nd |
| Anti-mumps IgG (AI) | neg | neg | neg | neg | neg | nd |
| Anti-rubella IgG (IU/ml) | neg | pos | pos | pos | pos | 9,6 (n:>10) |
| Lymphocyte subsets |  |  |  |  |  |  |
| CD3^+^ T-cell, (%) | 86.8 | 67.4 | 48.3 | 56.6 | 51.4 | 50.2 |
| CD3^+^ T-cell, count (/mm^3^) | 868 | 613 | 338 | 170 | 1494 | 803 |
| CD3^+^ CD4^+^ T, (%) | 34.4 | 26.5 | 31 | 34.8 | 23.6 | 14.58 |
| CD3^+^ CD4^+^ T, count (/mm^3^) | 344 | 241 | 217 | 104 | 682 | 233 |
| CD3^+^ CD8^+^ T, (%) | 52.6 | 32.8 | 17.2 | 19.9 | 27.4 | 36.6 |
| CD3^+^ CD8^+^ T, count (/mm^3^) | 526 | 298 | 120 | 60 | 791 | 585 |
| CD19^+^ B-cell, (%) | 5.58 | 14 | 35.3 | 8.97 | 35.5 | 22.3 |
| CD19^+^ B-cell, count (/mm^3^) | 58 | 127 | 247 | 27 | 1025 | 356 |
| CD16^+^56^+^ NK-cell, (%) | 0.88 | 15.6 | 7.8 | 17.2 | 7.46 | 11.8 |
| CD16^+^56^+^ NK-cell, count (/mm^3^) | 8.8 | 142 | 54.6 | 52 | 215 | 188 |
| Naive B-cell, (%) | 3.27 | NR | 80.9 | 89 | 60.3 | 82.4 |
| NS B-cell, (%) | 0 | NR | 17.5 | 5.86 | 5.98 | 2.17 |
| CS B-cell, (%) | 7.09 | NR | 0.2 | 1.67 | 11.09 | 2.11 |
| CD21^low^ CD38^low^ activated B, (%) | 13.64 | NR | 0.85 | 1.67 | 8.85 | 3.62 |
| CD3^+^ TCR^α/β^ cells, (%) | 91.3 | NR | 87.8 | 87.2 | 92.86 | 91.96 |
| CD3^+^ TCR^γ/δ^ cells, (%) | 5.87 | NR | 4.5 | 5.66 | 3.3 | 2.57 |
| Recent thymic emigrants, (%) | 0.51 | NR | 0.5 | 0.1 | 2.37 | 0.7 |
| CD4^+^ CD45RA^+^ CCR7^+^ T-cell, (%) | 1.1 | NR | 1.7 | 0.92 | 0.93 | 1.16 |
| CD4 ^+^ CD45RA^-^ CCR7^+^ T-cell, (%) | 9.4 | NR | 73.3 | 87.3 | 77.9 | 20.6 |
| CD4^+^ CD45RA^-^ CCR7^-^ T-cell, (%) | 86.2 | NR | 24.1 | 11.7 | 20.4 | 77.1 |
| CD4^+^ CD45RA^+^ CCR7^-^ T-cell, (%) | 3.3 | NR | 0.81 | 0.05 | 0.75 | 1.09 |
| CD8^+^ CD45RA^+^ CCR7^+^ T-cell, (%) | 3.03 | NR | 20.2 | 23.2 | 6.4 | 2.95 |
| CD8^+^ CD45RA^-^ CCR7^+^ T-cell, (%) | 1.09 | NR | 55.7 | 40 | 74.6 | 13.7 |
| CD8^+^ CD45RA^-^ CCR7^-^ T-cell, (%) | 23.17 | NR | 11.7 | 14.2 | 11.9 | 67 |
| CD8^+^ CD45RA^+^ CCR7^-^ T-cell, (%) | 72.7 | NR | 12.3 | 22.45 | 7.04 | 16.3 |

**CS:** Class switched, IVIG: Intravenous immunoglobulin, **NS:** Non-class switched, (-): Not done. Abnormal values are indicated in the parenthesis. NR: Could not be retrieved

***:** Under IVIG
