# Supplementary material for: DIAPH1-Deficiency is Associated with Major T, NK and ILC Defects in Humans
Source: J Clin Immunol. 2024 Aug 9;44(8):175. doi: 10.1007/s10875-024-01777-8 (PMC11315734; doi:10.1007/s10875-024-01777-8)
Supplement: Supplementary file 6 — Supplementary Material 6 [file 10875_2024_1777_MOESM6_ESM.docx]

**TABLE S5.** List of DIAPH1 mutations studied do this date.

| **Nucleotide** | **Amino acid** | **Exon** | **Protein domain** | **Variation**  **type** | **Disease** | **Origin** | **Families** | **Patients** | **Reference** |
| --- | --- | --- | --- | --- | --- | --- | --- | --- | --- |
| c.663G>C | p.Leu221Phe | 7 | DID | Missense | DFNA1 | Japan | 1 | 1 | 2016. Iwasa et al. |
| c.684+1G>A |  | Intronic  (7-8) | DID | Splicing | SCBMS | Finland | 5 | 5 | 2021. Kaustio et al. |
| c.692T>C | p.Ile231Thr | 8 | DID | Missense | DFNA1 | Czech Republic | 1 | 2 | 2020. Brozkova et al. |
| c.793G>T | p.Ala265Ser | 8 | DID | Missense | DFNA1 | Korea | 1 | 4 | 2019. Kim et al. |
| **c.1051C>T** (NM_005219.4, isoform 1) | **p.R351*** | 11 | **FH3/DID** | **Nonsense** | **SCBMS** | **Turkey** | **5** | **5** | **This study**  **(Azizoglu et al 2024)** |
| **c.964C>T,** (NM_001079812.3, isoform 2) | **p. Arg322***  (corresponds to p.Arg331* in isoform 1\AZ |  | **FH3/DID** | **Nonsense** | **SCBMS** | **Turkey** | **1** | **1** | **This study**  **(Azizoglu et al 2024)** |
| c.1589T>G | p.Ile530Ser | 15 | CC | Missense | DFNA1 | Korea | 1 | 3 | 2016. Kang et al. |
| c.2032C>T | p.Pro678Ser | 16 | FH1 | Missense | DFNA1 | Korea | 1 | 4 | 2012. Baek et al. |
| c.2332C4T | p.Gln778X | 16 | FH2 | Nonsense | SCBMS | Saudi Arabia | 1 | 5 | 2014. Sencicek et al. |
| c.2482+1G>T |  | 18 | FH2 | Splicing | DFNA1 | Belgium | 2 | 2 | 2016. Sommen at al. |
| c.2769delT | p.Phe923fs* | 21 | FH2 | Frameshift | SCBMS | Oman | 2 | 2 | 2021. Kaustio et al. |
|  |  |  |  |  |  |  | 3 | 1 | 2016. Al-Maawali et al. |
| c.3145C>T | p.Arg1049X | 23 | FH2 | Nonsense | SCBMS | United Arab Emirates | 1 | 1 | 2016. Al-Maawali et al. |
| c.3551_3552del | p.Glu1184AlafsTer11 | 26 | FH2+DAD | Frameshift | DFNA1 | China | 1 | 16 | 2020. Wu et al. |
|  | p.Glu1192_Gln1220del | 27 | FH2+DAD | Deletion | DFNA1+Thrombocytopenia | England | 5 | 16 | 2018. Westbury et al. |
| c.3589G>A | p.Gly1197Ser | 27 | DAD | Missense | DFNA1 | USA | 1 | 1 | 2013. Shearer et al. |
| c.3610C>T | p.Arg1204Tera | 27 | DAD | Nonsense | DFNA1 | Japan | 2 | 4/2 | 2016.Ueyama et al. |
| c.3771_3772delAG | p.Ala1210Glyfs*31 | 27 | DAD | Frameshift | DFNA1+Thrombocytopenia | England | 5 | 16 | 2018. Westbury et al. |
| c.3624_3625del | p.Ala1210SerfsTer31 | 27 | DAD | Frameshift | DFNA1+  Thrombocytopenia | Germany | 1 | 5 | 2016. Neuhaus et al. |
| c.3637C>T | p.Arg1213X | 27 | DAD | Nonsense | DFNA1+Thrombocytopenia | Japan | 1 | 1 | 2016. Iwasa et al. |
|  |  |  |  |  |  | England  /France | 2 | 5/3 | 2016. Stritt et al. |
|  |  |  |  |  |  | Germany | 2 | 5/4 | 2017. Neuhaus et al. |
|  |  |  |  |  |  | Japanese | 1 | 12 | 2017. Ganaha et al. |
|  |  |  |  |  |  | Brazil | 2 | 2 | 2018. Bastida et al |
|  |  |  |  |  |  | Australia | 1 | 2 | 2020. Rabbolini et al. |
|  |  |  |  |  |  | Caucasian | 1 | 5 | 2021. Karki et al. |
| c.3661+1G>T /IVS27 ds G-T +1 | p.Ala1221ValfsTer22 | 27 | DAD | Frameshift | DFNA1 | Costa Rica | 1 | 78 | 1997. Lynch, et al. |
|  | CNV gain and loss |  |  |  | DFNA1 | China |  | 29 | 2014. Ji et al. |

**REFERENCES**

Iwasa YI, Nishio SY, Usami SI. Comprehensive Genetic Analysis of Japanese Autosomal Dominant Sensorineural Hearing Loss Patients. PLoS One. 2016 Dec 2;11(12):e0166781.

Kaustio M, Nayebzadeh N, Hinttala R, Tapiainen T, Åström P, Mamia K, et al. Loss of DIAPH1 causes SCBMS, combined immunodeficiency, and mitochondrial dysfunction. J Allergy Clin Immunol. 2021 Aug;148(2):599-611. doi: 10.1016/j.jaci.2020.12.656. Epub 2021 Mar 1. Erratum in: J Allergy Clin Immunol. 2021 Dec;148(6):1603.

Safka Brozkova D, Poisson Marková S, Mészárosová AU, Jenčík J, Čejnová V, Čada Z, et al. Spectrum and frequencies of non GJB2 gene mutations in Czech patients with early non-syndromic hearing loss detected by gene panel NGS and whole-exome sequencing. Clin Genet. 2020 Dec; 98(6):548-554.

Kim BJ, Ueyama T, Miyoshi T, Lee S, Han JH, Park HR, et al. Differential disruption of autoinhibition and defect in assembly of cytoskeleton during cell division decide the fate of human DIAPH1-related cytoskeletopathy. J Med Genet. 2019 Dec;56(12):818-827.

Kang TH, Baek JI, Sagong B, Park HJ, Park CI, Lee KY, Kim UK. A novel missense variant in the DIAPH1 gene in a Korean family with autosomal dominant nonsyndromic hearing loss. Genes Genet Syst. 2017 Apr 4;91(5):289-292.

Baek JI, Oh SK, Kim DB, Choi SY, Kim UK, Lee KY, Lee SH. Targeted massive parallel sequencing: the effective detection of novel causative mutations associated with hearing loss in small families. Orphanet J Rare Dis. 2012 Sep 3;7:60.

Ercan-Sencicek AG, Jambi S, Franjic D, Nishimura S, Li M, El-Fishawy P, et al. Homozygous loss of DIAPH1 is a novel cause of microcephaly in humans. Eur J Hum Genet. 2015 Feb;23(2):165-72.

Sommen M, Schrauwen I, Vandeweyer G, Boeckx N, Corneveaux JJ, van den Ende J, et al. DNA Diagnostics of Hereditary Hearing Loss: A Targeted Resequencing Approach Combined with a Mutation Classification System. Hum Mutat. 2016 Aug;37(8):812-9.

Al-Maawali A, Barry BJ, Rajab A, El-Quessny M, Seman A, Coury SN, Barkovich AJ, et al. Novel loss-of-function variants in DIAPH1 associated with syndromic microcephaly, blindness, and early onset seizures. Am J Med Genet A. 2016 Feb;170A(2):435-440.

Wu K, Wang H, Guan J, Lan L, Zhao C, Zhang M, Wang D, Wang Q. A novel variant in diaphanous homolog 1 (DIAPH1) as the cause of auditory neuropathy in a Chinese family. Int J Pediatr Otorhinolaryngol. 2020 Jun;133:109947.

Westbury SK, Downes K, Burney C, Lozano ML, Obaji SG, Toh CH, Sevivas T, et al. Phenotype description and response to thrombopoietin receptor agonist in DIAPH1-related disorder. Blood Adv. 2018 Sep 25;2(18):2341-2346.

Shearer AE, Black-Ziegelbein EA, Hildebrand MS, Eppsteiner RW, Ravi H, Joshi S,et al. Advancing genetic testing for deafness with genomic technology. J Med Genet. 2013 Sep;50(9):627-34.

Ueyama T, Ninoyu Y, Nishio SY, Miyoshi T, Torii H, Nishimura K, Sugahara K, et al. Constitutive activation of DIA1 (DIAPH1) via C-terminal truncation causes human sensorineural hearing loss. EMBO Mol Med. 2016 Nov 2;8(11):1310-1324.

Neuhaus C, Lang-Roth R, Zimmermann U, Heller R, Eisenberger T, Weikert M, et al. Extension of the clinical and molecular phenotype of DIAPH1-associated autosomal dominant hearing loss (DFNA1). Clin Genet. 2017 Jun;91(6):892-901.

Stritt S, Nurden P, Turro E, Greene D, Jansen SB, Westbury SK, et al. A gain-of-function variant in DIAPH1 causes dominant macrothrombocytopenia and hearing loss. Blood. 2016 Jun 9;127(23):2903-14.

Ganaha A, Kaname T, Shinjou A, Chinen Y, Yanagi K, Higa T, et al. Progressive macrothrombocytopenia and hearing loss in a large family with DIAPH1 related disease. Am J Med Genet A. 2017 Oct;173(10):2826-2830.

Bastida JM, Lozano ML, Benito R, Janusz K, Palma-Barqueros V, Del Rey M, et al. Introducing high-throughput sequencing into mainstream genetic diagnosis practice in inherited platelet disorders. Haematologica. 2018 Jan;103(1):148-162.

Rabbolini D, Connor D, Morel-Kopp MC, Donikian D, Kondo M, Chen W,et al. Sydney Platelet Group. An integrated approach to inherited platelet disorders: results from a research collaborative, the Sydney Platelet Group. Pathology. 2020 Feb;52(2):243-255.

Karki NR, Ajebo G, Savage N, Kutlar A. DIAPH1 Mutation as a Novel Cause of Autosomal Dominant Macrothrombocytopenia and Hearing Loss. Acta Haematol. 2021;144(1):91-94.

Lynch ED, Lee MK, Morrow JE, Welcsh PL, León PE, King MC. Nonsyndromic deafness DFNA1 associated with mutation of a human homolog of the Drosophila gene diaphanous. Science. 1997 Nov 14;278(5341):1315-8.

Ji H, Lu J, Wang J, Li H, Lin X. Combined examination of sequence and copy number variations in human deafness genes improves diagnosis for cases of genetic deafness. BMC Ear Nose Throat Disord. 2014 Sep 10;14:9.
