# Supplementary material for: DIAPH1-Deficiency is Associated with Major T, NK and ILC Defects in Humans
Source: J Clin Immunol. 2024 Aug 9;44(8):175. doi: 10.1007/s10875-024-01777-8 (PMC11315734; doi:10.1007/s10875-024-01777-8)
Supplement: Supplementary file 7 — Supplementary Material 7 [file 10875_2024_1777_MOESM7_ESM.docx]

**TABLE S6.** Primers

| PrImers | FW 5’ to 3’ | RV 5’ to 3’ |
| --- | --- | --- |
| *DIAPH1* | CGACGGCGGCAAATCTAAG | TGAGCAGAATTGGGCTTTTCC |
| 18S | GTAACCCGTTGAACCCCATT | CCATCCAATCGGTAGTAGCG |
