# Supplementary figures and images for: DIAPH1-Deficiency is Associated with Major T, NK and ILC Defects in Humans
Source: J Clin Immunol. 2024 Aug 9;44(8):175. doi: 10.1007/s10875-024-01777-8 (PMC11315734; doi:10.1007/s10875-024-01777-8)

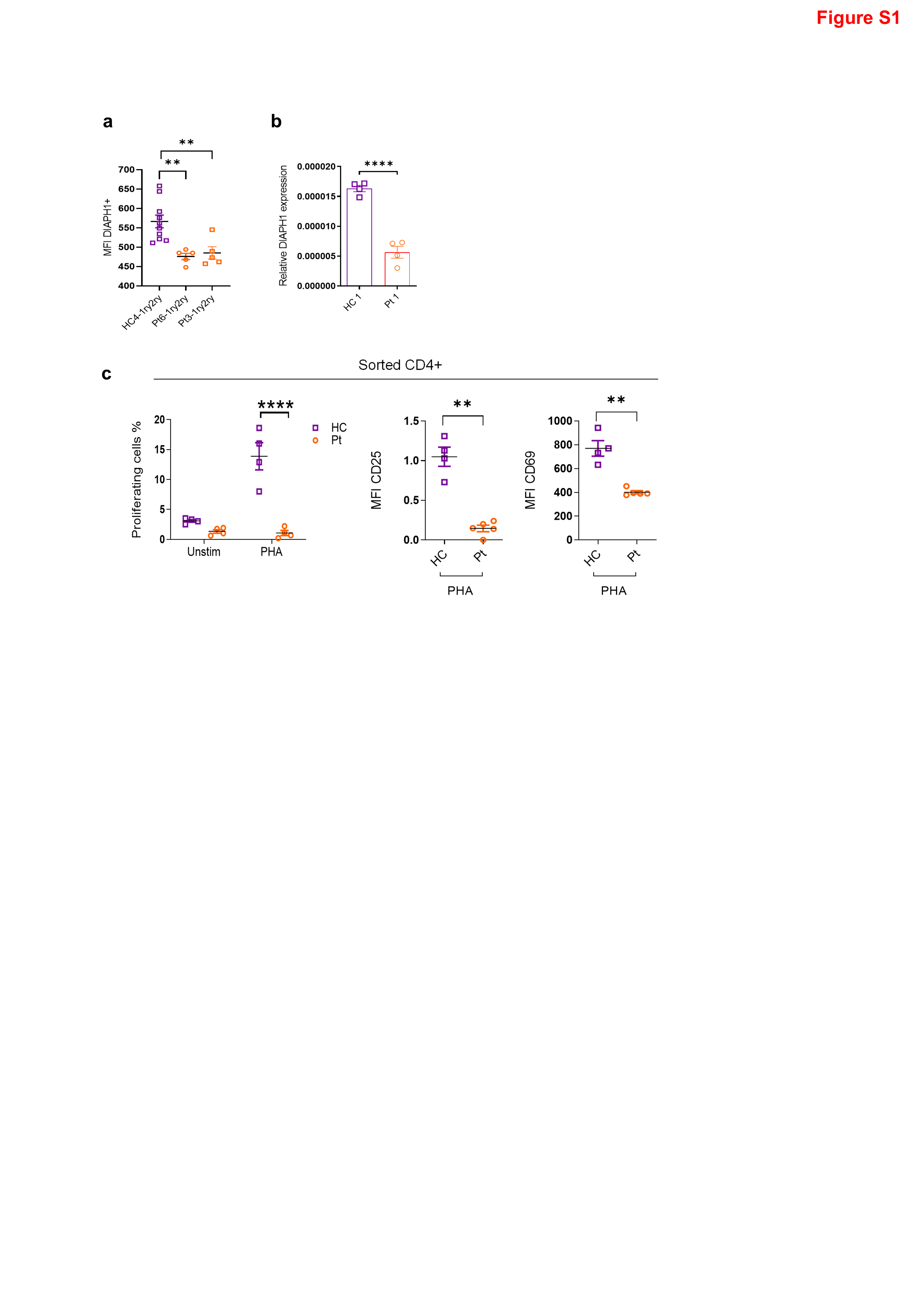

Supplement: Supplementary file 8 — Supplementary Material 8 [file 10875_2024_1777_MOESM8_ESM.png]

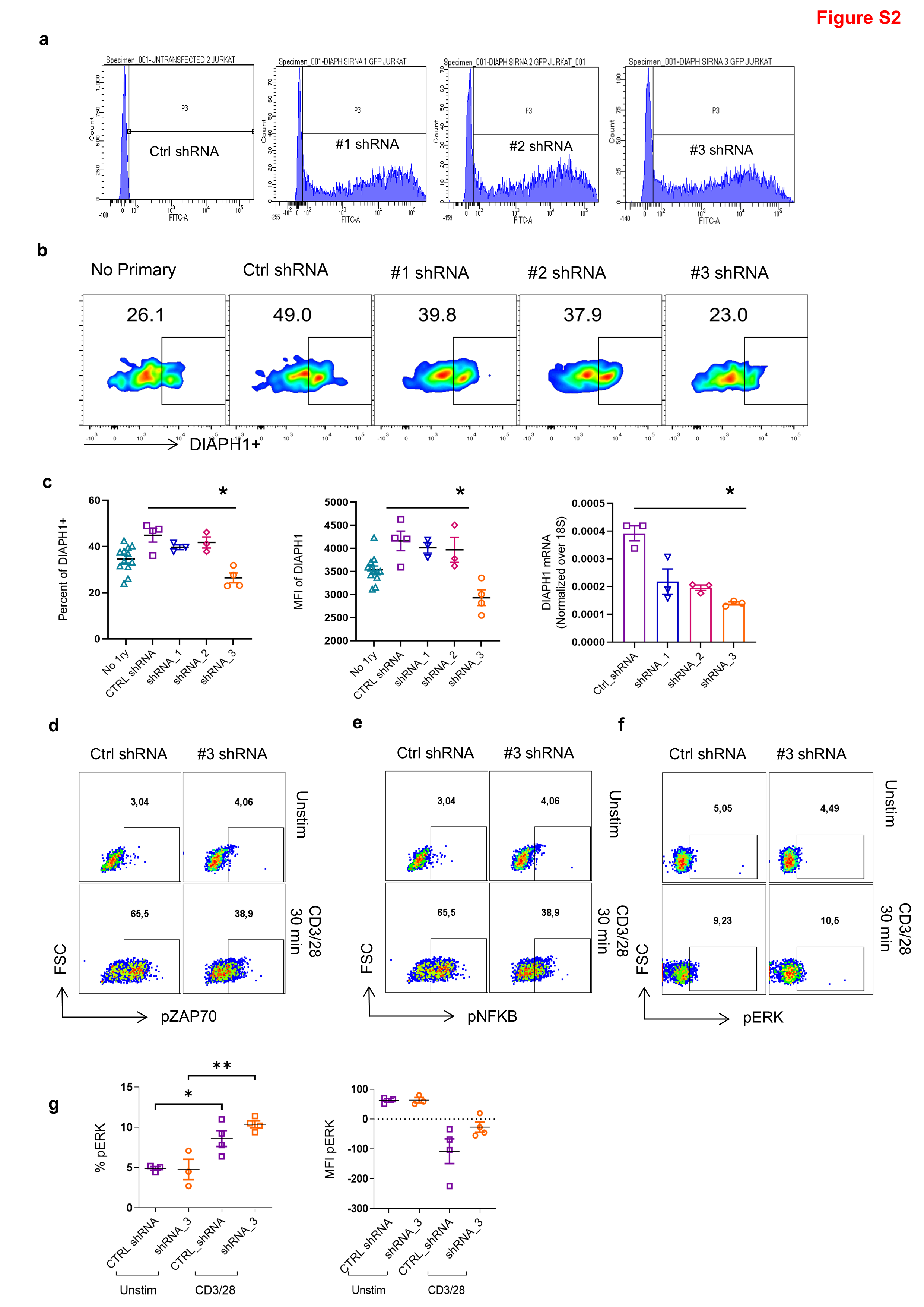

Supplement: Supplementary file 9 — Supplementary Material 9 [file 10875_2024_1777_MOESM9_ESM.png]

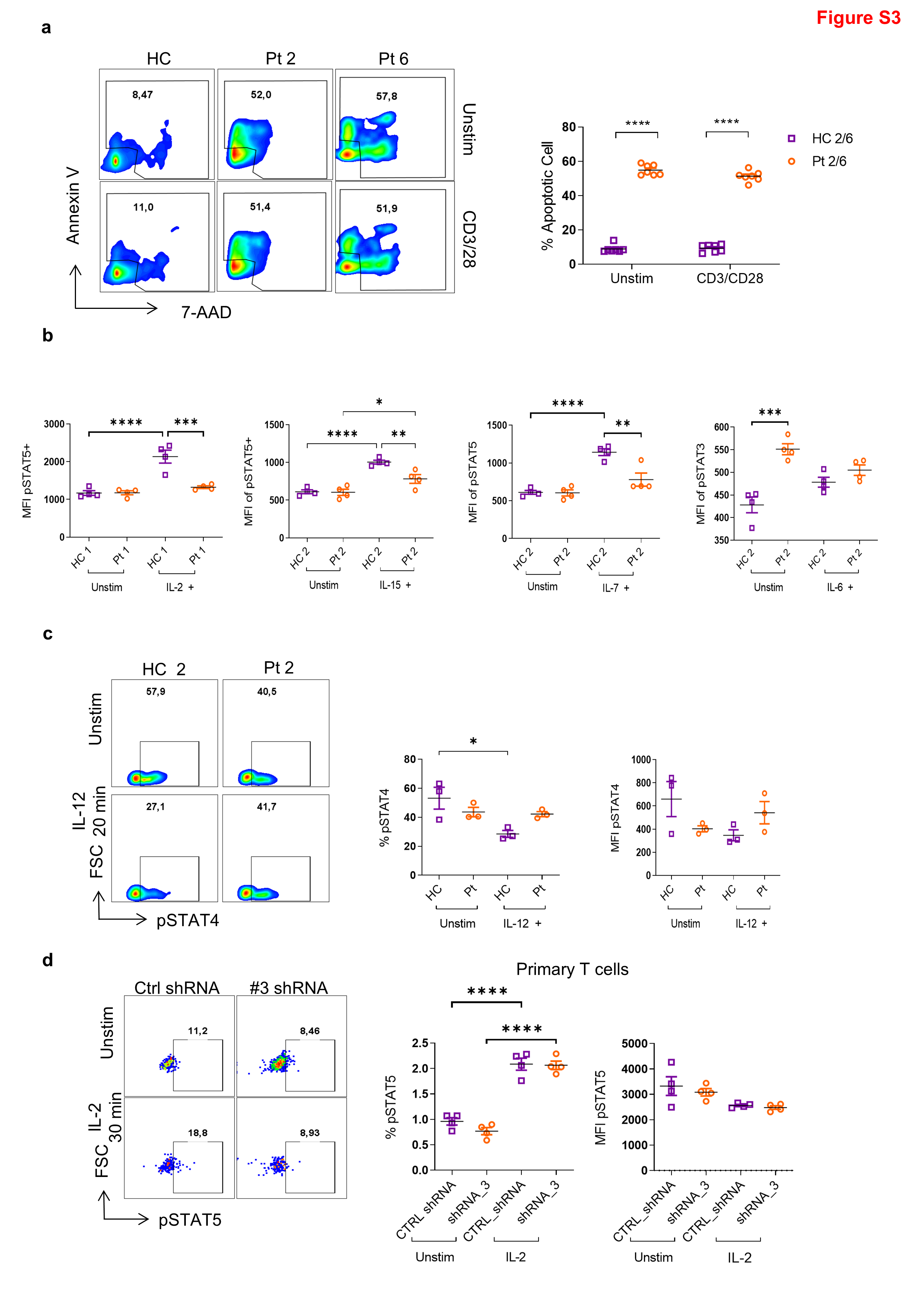

Supplement: Supplementary file 10 — Supplementary Material 10 [file 10875_2024_1777_MOESM10_ESM.png]

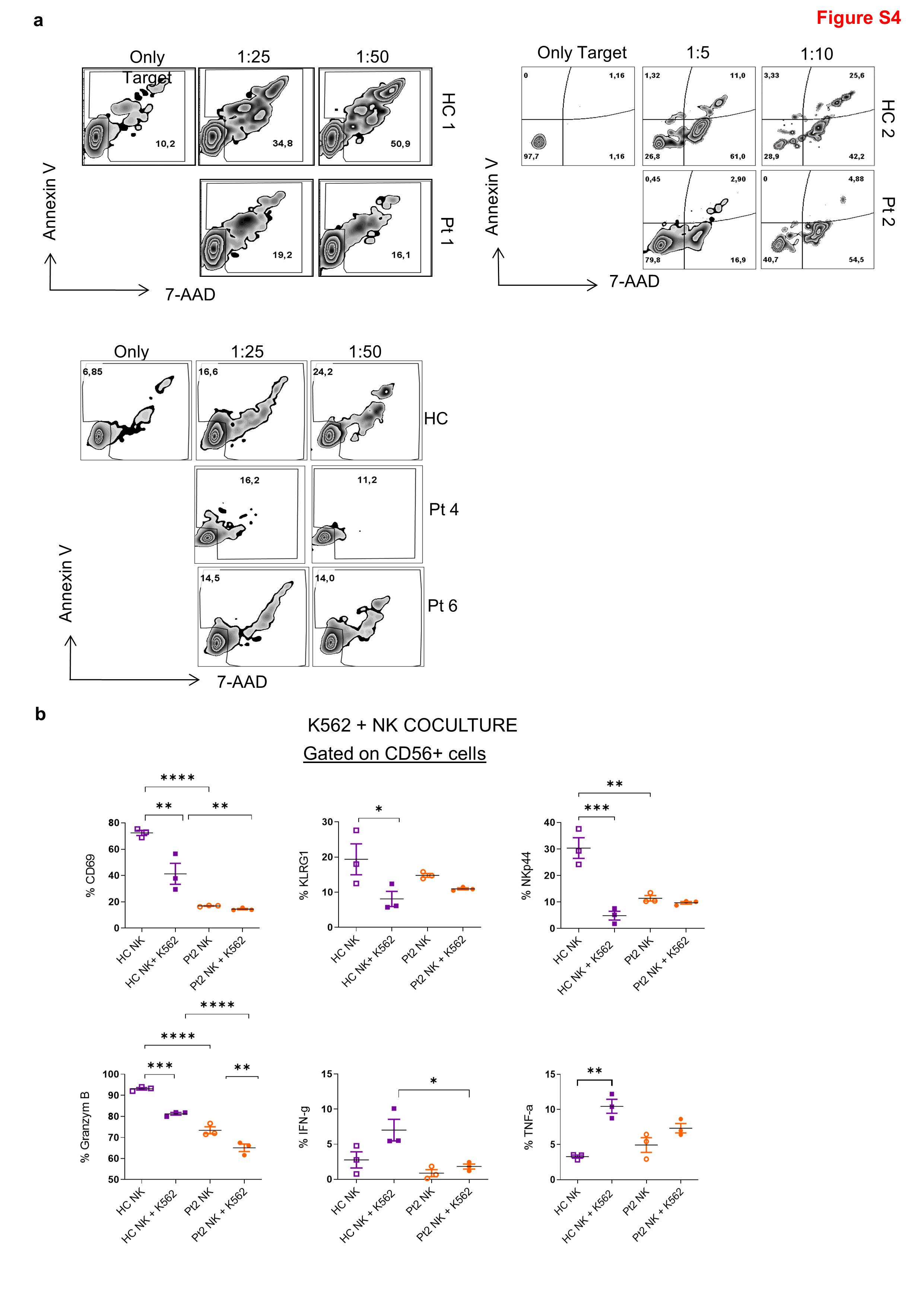

Supplement: Supplementary file 11 — Supplementary Material 11 [file 10875_2024_1777_MOESM11_ESM.png]

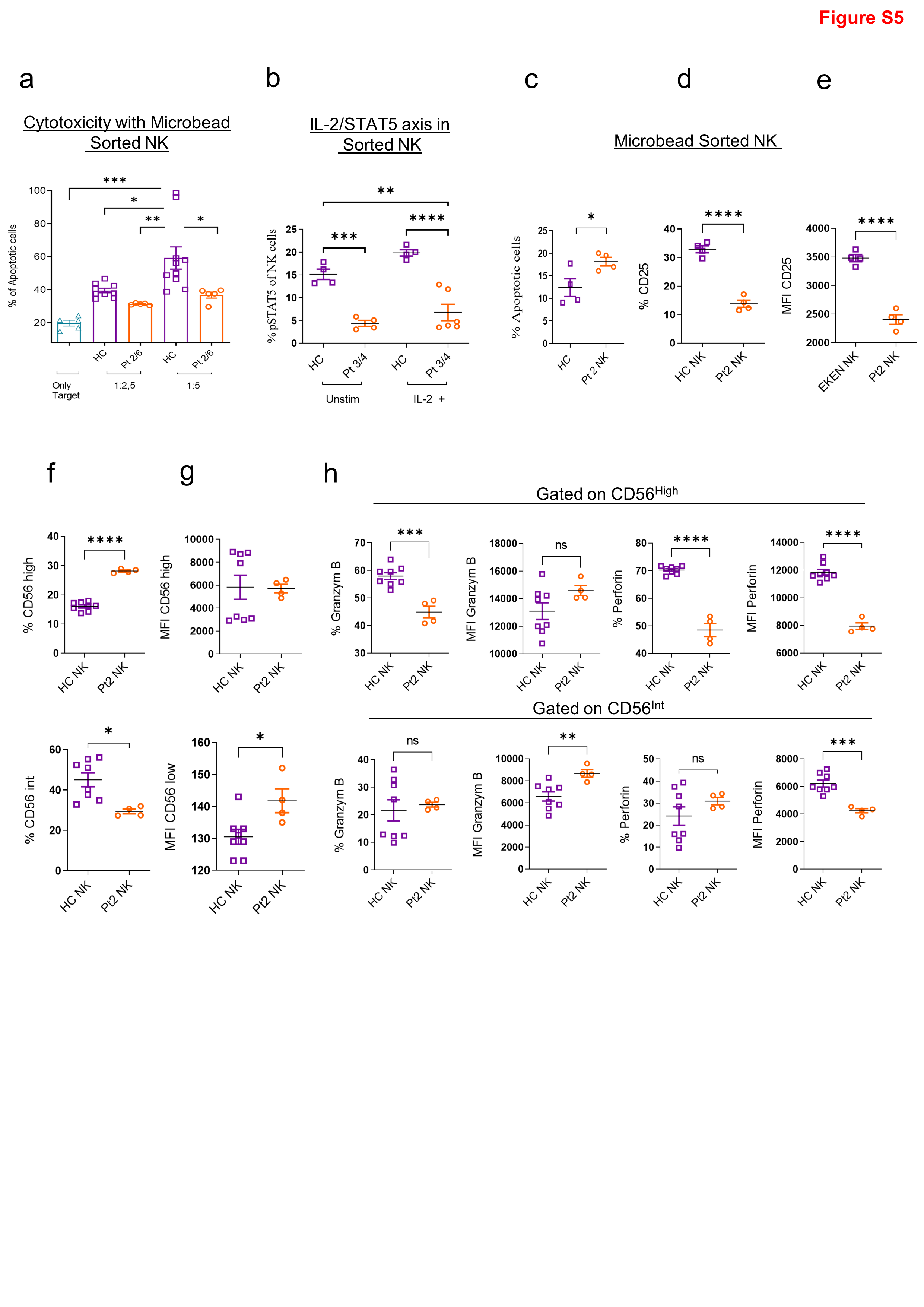

Supplement: Supplementary file 12 — Supplementary Material 12 [file 10875_2024_1777_MOESM12_ESM.png]
